# BRCA1 Mutation‐Recognizing Tetrahedral Framework Nucleic Acid Nanoplatform Enables Coordinated PARP/WEE1 Inhibition and STING‐Associated Antitumor Immunity in Ovarian Cancer

**DOI:** 10.1002/advs.78457

**Published:** 2026-10-28

**Authors:** Chenyi Wang, Mengying Yao, Jinhui Xie, Dingjie Wang, Yashi Shi, Xiaodan Mao, Chunhua Lu, Pengming Sun

**Affiliations:** ^1^ Laboratory of Gynecologic Oncology College of Clinical Medicine for Obstetrics & Gynecology and Pediatrics Fujian Maternity and Child Health Hospital Fujian Medical University Fuzhou China; ^2^ Fujian Clinical Research Center for Gynecological Oncology Fujian Maternity and Child Health Hospital (Fujian Obstetrics and Gynecology Hospital) Fuzhou China; ^3^ Fujian Key Laboratory of Women and Children's Critical Diseases Research Fuzhou China; ^4^ School of Medicine Fuzhou University Fuzhou China; ^5^ MOE Key Laboratory For Analytical Science of Food Safety and Biology Fujian Key Laboratory of Analysis and Testing Technology For Food Safety and Health College of Chemistry Fuzhou University Fuzhou China

**Keywords:** BRCA1 mutation, ovarian cancer, PARP/WEE1 inhibitor, STING signaling, synthetic lethality

## Abstract

Improving therapeutic efficacy and antitumor immune responsiveness in BRCA1‐mutant ovarian cancer remains challenging. Here, we developed a BRCA1 mutation‐guided tetrahedral framework nucleic acid nanosystem, FBOA‐tFNA, for coordinated intracellular delivery of the PARP inhibitor olaparib and the WEE1 inhibitor adavosertib. After folate receptor‐mediated uptake, FBOA‐tFNA recognizes BRCA1 mutant RNA through toehold‐mediated strand displacement, enabling mutation‐selective beacon activation, while drug release is independently regulated by reduction‐responsive disulfide cleavage. Coordinated PARP/WEE1 inhibition amplified DNA damage, impaired G2/M checkpoint protection, and promoted tumor‐cell killing in BRCA1‐mutant ovarian cancer models. Resulting DNA damage‐associated intracellular dsDNA signals activated STING‐associated signaling, type I interferon responses, immunogenic cell death (ICD)‐associated stress, dendritic‐cell maturation, and CD8^+^ T‐cell activation. Engineered patient‐derived ovarian cancer organoids expressing a BRCA1^2594delC^‐containing transcript further supported the mutation‐selective recognition and therapeutic potential of FBOA‐tFNA ex vivo. In vivo, FBOA‐tFNA showed tumor‐associated fluorescence accumulation, BRCA1 mutant‐transcript recognition‐associated beacon activation, enhanced antitumor efficacy, and preliminary repeated‐dose tolerability. Moreover, FBOA‐tFNA enhanced antitumor immune responses and improved the response to anti‐PD‐1 therapy, accompanied by increased effector and memory‐like T‐cell populations. This programmable DNA nanoplatform provides a personalized combination strategy for coordinated DNA‐damage therapy and immunomodulation in BRCA1‐mutant ovarian cancer.

## Graphical abstract

BRCA1 mutation‐responsive FBOA‐tFNA selectively recognizes mutant transcripts and coordinates reduction‐responsive intracellular delivery of olaparib and adavosertib, amplifying DNA damage and weakening G2/M checkpoint protection. These effects are accompanied by STING‐associated immune activation and stronger antitumor immunity, while sequential anti‐PD‐1 treatment further improves tumor control and promotes memory‐associated T‐cell responses.

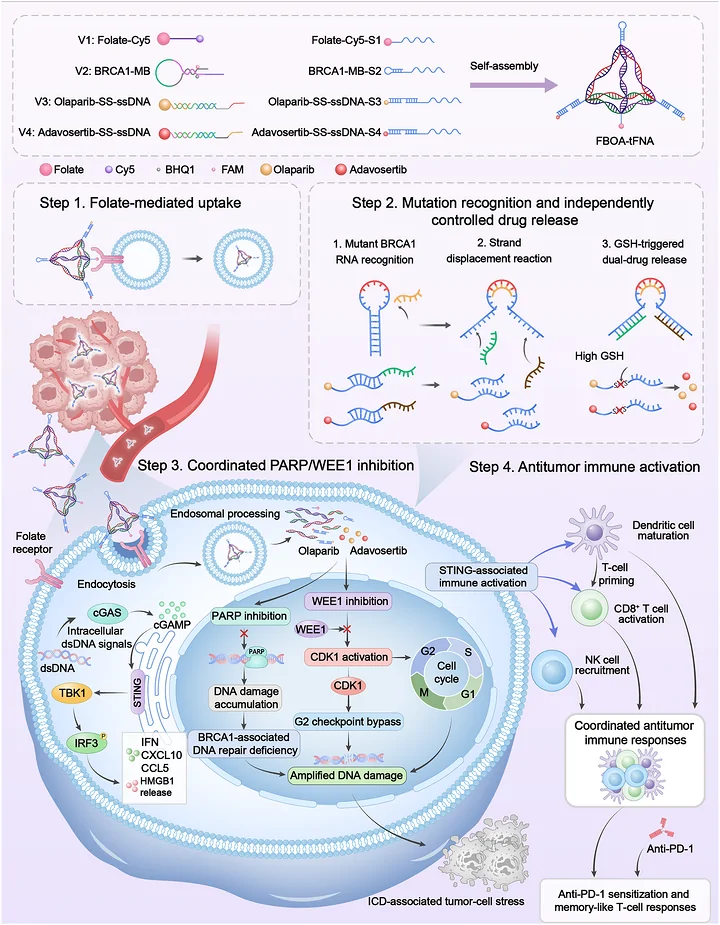

## 1 Introduction

Ovarian cancer remains one of the most aggressive malignancies in women, characterized by high recurrence rates, rapid progression, and limited therapeutic options. High‐grade serous ovarian cancer is one of the most prevalent and lethal histological subtypes of ovarian cancer. Due to its insidious onset and lack of early symptoms, most patients are diagnosed at advanced stages, accompanied by extensive peritoneal dissemination, malignant ascites formation, and high postoperative recurrence risk [1, 2, 3]. The complex metastatic patterns of advanced ovarian cancer, including lymphatic dissemination and lymph node involvement, further complicate clinical management [4]. Although cytoreductive surgery combined with platinum‐based chemotherapy has significantly improved the prognosis of some patients, recurrent and drug‐resistant disease remains the leading cause of therapeutic failure and mortality [5, 6]. Related homologous recombination deficiency (HRD)‐associated malignancies, including triple‐negative breast cancer (TNBC), also exhibit limited therapeutic options and aggressive clinical behavior in selected molecular contexts [7, 8, 9]. Nevertheless, the present study focuses on ovarian cancer, in which homologous recombination repair deficiency, particularly associated with BRCA1/2 abnormalities, represents a critical molecular vulnerability [10, 11]. Tumors harboring BRCA mutations or homologous recombination deficiency are initially sensitive to platinum agents and poly(ADP‐ribose) polymerase inhibitors (PARPi); however, under sustained therapeutic pressure, tumor cells can acquire resistance through restoration of homologous recombination, replication fork stabilization, enhanced drug efflux, or rewiring of DNA damage response pathways [11, 12]. Therefore, precisely amplifying DNA damage in BRCA1‐mutant tumor cells while minimizing excessive toxicity to normal tissues remains a central challenge for improving therapeutic efficacy and limiting checkpoint‐mediated tolerance [13].

PARP inhibitors have revolutionized the therapeutic landscape of BRCA‐mutant ovarian and breast cancers through the mechanism of synthetic lethality [1, 12, 14]. Nevertheless, PARPi monotherapy is often insufficient to eradicate heterogeneous tumor cell populations, as subsets of tumor cells may survive by relying on cell‐cycle checkpoints to tolerate replication stress and DNA damage [13]. WEE1 kinase serves as a critical regulator of the G2/M checkpoint, and inhibition of WEE1 forces cells harboring DNA damage to prematurely enter mitosis, thereby inducing mitotic catastrophe and enhancing tumor cell killing [15, 16]. Accordingly, PARPi and WEE1 inhibitors exhibit strong mechanistic complementarity. However, conventional combination administration suffers from asynchronous pharmacokinetics, heterogeneous intratumoral distribution, and cumulative systemic toxicities, which restrict the synergistic interaction of the two agents within the same tumor cell and therapeutic time window [17].

Beyond directly inducing tumor cell death, DNA damage‐based therapies can also promote antitumor immune responses. Accumulation of unrepaired DNA damage and DNA damage‐associated nucleic acid stress can activate the cyclic GMP‐AMP synthase‐stimulator of interferon genes (cGAS‐STING) pathway, promoting type I interferon production, chemokine secretion, dendritic cell maturation, and T‐cell recruitment [18, 19]. Recent advances in nanomedicine have further highlighted the potential of nanoplatform‐mediated STING modulation for enhancing antitumor immunity and cancer immunotherapy [20]. Previous studies have demonstrated that PARP inhibition can induce STING‐dependent antitumor immunity, while combined PARP and WEE1 inhibition further enhances DNA damage‐associated immune activation [17]. However, ovarian cancer is frequently characterized by an immunologically “cold” tumor microenvironment with limited responsiveness to immune checkpoint blockade alone, a feature also observed in several related HRD‐associated malignancies [9, 21, 22]. Therefore, an ideal therapeutic strategy should not only potentiate synthetic lethality but also promote immunogenic tumor‐cell stress and antitumor immune activation, thereby enhancing tumor sensitivity to immune checkpoint blockade therapy. Recent studies have demonstrated that STING‐associated immune activation can be integrated with therapeutic strategies to improve responses to immune checkpoint blockade [23].

Tetrahedral framework nucleic acids (tFNAs) provide a highly programmable nanoplatform for addressing these challenges. Owing to their controllable size, structural stability, excellent biocompatibility, and site‐specific functionalization capability, tFNAs have been widely utilized for integrating targeting ligands, molecular beacons, strand displacement modules, and therapeutic payloads [24, 25, 26]. Toehold‐mediated strand displacement reactions enable sequence‐specific molecular recognition and conditional structural reconfiguration, making them particularly suitable for constructing mutation‐responsive recognition systems coupled with environmentally responsive drug‐delivery modules [27, 28]. However, current tFNA‐based delivery systems have not yet fully integrated BRCA1 mutant‐transcript recognition with coordinated PARP/WEE1 dual‐drug delivery, nor have they been extensively applied to coordinate DNA damage amplification, cell‐cycle collapse, and antitumor immune activation [17, 24].

In the present study, we developed a BRCA1 mutation‐responsive tetrahedral framework nucleic acid nanosystem, termed FBOA‐tFNA, to achieve coordinated intracellular delivery of PARP and WEE1 inhibitors. This system integrates a folate‐mediated targeting module, a BRCA1 mutation‐transcript‐responsive molecular beacon, and disulfide‐linked olaparib and adavosertib modules on a single tFNA scaffold. Cy5‐ and fluorescein amidite (FAM)/ Black Hole Quencher 1 (BHQ1)‐labeled variants were used for fluorescence‐based tracking and mutation‐responsive activation studies. Following folate receptor‐mediated endocytosis, FBOA‐tFNA preferentially accumulates in tumor cells and recognizes BRCA1 mutant RNA to trigger toehold‐mediated strand displacement. Meanwhile, the glutathione‐rich intracellular tumor environment promotes disulfide bond cleavage, supporting reduction‐responsive intracellular drug release. We hypothesized that this mutation‐responsive recognition combined with an independently controlled reduction‐responsive dual‐drug delivery strategy would improve the intracellular coordination of PARP and WEE1 inhibition within BRCA1‐mutant tumor cells. Specifically, FBOA‐tFNA amplifies DNA damage through PARP inhibition while simultaneously weakening G2/M checkpoint protection via WEE1 inhibition, forcing damaged cells into aberrant mitosis and inducing enhanced tumor‐cell killing and ICD‐associated stress. Furthermore, sustained DNA damage and DNA damage‐associated intracellular double‐stranded DNA (dsDNA) signals are expected to be associated with STING‐associated immune signaling, type I interferon and chemokine production, dendritic cell maturation, CD8^+^ T‐cell responses, and NK‐cell infiltration, thereby enhancing antitumor immune responses and potentially sensitizing tumors to anti‐PD‐1 therapy. Collectively, this study presents an intelligent DNA nanotherapeutic strategy that integrates precise BRCA1 mutant‐transcript recognition, coordinated PARP/WEE1 dual‐drug delivery, reduction‐responsive intracellular drug release, DNA damage amplification, and antitumor immune activation, thereby providing a novel personalized combinational strategy for BRCA1‐mutant ovarian cancer.

## 2 Results

### 2.1 Rational Design, Precise Characterization, and Validation of the Mutation‐Responsive Recognition of FBO‐tFNA

Taking advantage of the structural stability, controllable size, programmable functionalization, and biocompatibility of tetrahedral framework nucleic acids (tFNAs), we constructed a BRCA1 mutation‐responsive nanodelivery system, termed FBO‐tFNA [24, 29, 30]. FBO‐tFNA employed a three‐vertex functionalization strategy: V1 carried a folate‐mediated targeting module; V2 incorporated a BRCA1 2594delC‐responsive molecular beacon for sequence‐specific mutant RNA recognition and beacon activation [31, 32, 33], and V3 contained a disulfide‐linked olaparib prodrug module (Olaparib‐SS‐ssDNA) for reduction‐responsive drug release. V4 was retained to preserve scaffold stability (Figure 1A and Figures S1–S4) [34]. For fluorescence‐based uptake and activation studies, Cy5 was incorporated into the folate module and the molecular beacon was labeled with FAM/BHQ1, integrating folate receptor‐α (FR‐α, encoded by FOLR1)‐mediated internalization, mutation‐responsive molecular recognition, and reduction‐responsive drug liberation as distinct functional processes within one nanostructure [28, 35]. Four functionalized DNA strands were assembled at equimolar ratios by one‐pot gradient annealing [36]. Native polyacrylamide gel electrophoresis (PAGE) showed stepwise mobility retardation after addition of each module, confirming successful assembly (Figure 1B). Transmission electron microscopy (TEM) showed comparable tetrahedral‐like projected morphologies of bare tFNA and FBO‐tFNA, while dynamic light scattering (DLS) showed a hydrodynamic diameter of 15.9 ± 4.2 nm, a PDI of 0.143 ± 0.006, and a zeta potential of −25.3 ± 0.4 mV (Figure 1C–F), indicating homogeneity and colloidal stability. Olaparib loading efficiency was approximately 80.5%, with drug incorporation approaching one molecule per tFNA at higher input equivalents (Figure S5A–C,G,H). FBO‐tFNA maintained structural integrity for 12 h under DNase I exposure or in 10% fetal bovine serum (FBS), as supported by quantitative densitometric analysis of the corresponding PAGE bands (Figure 1G–J), demonstrating resistance to degradation.

**FIGURE 1:**
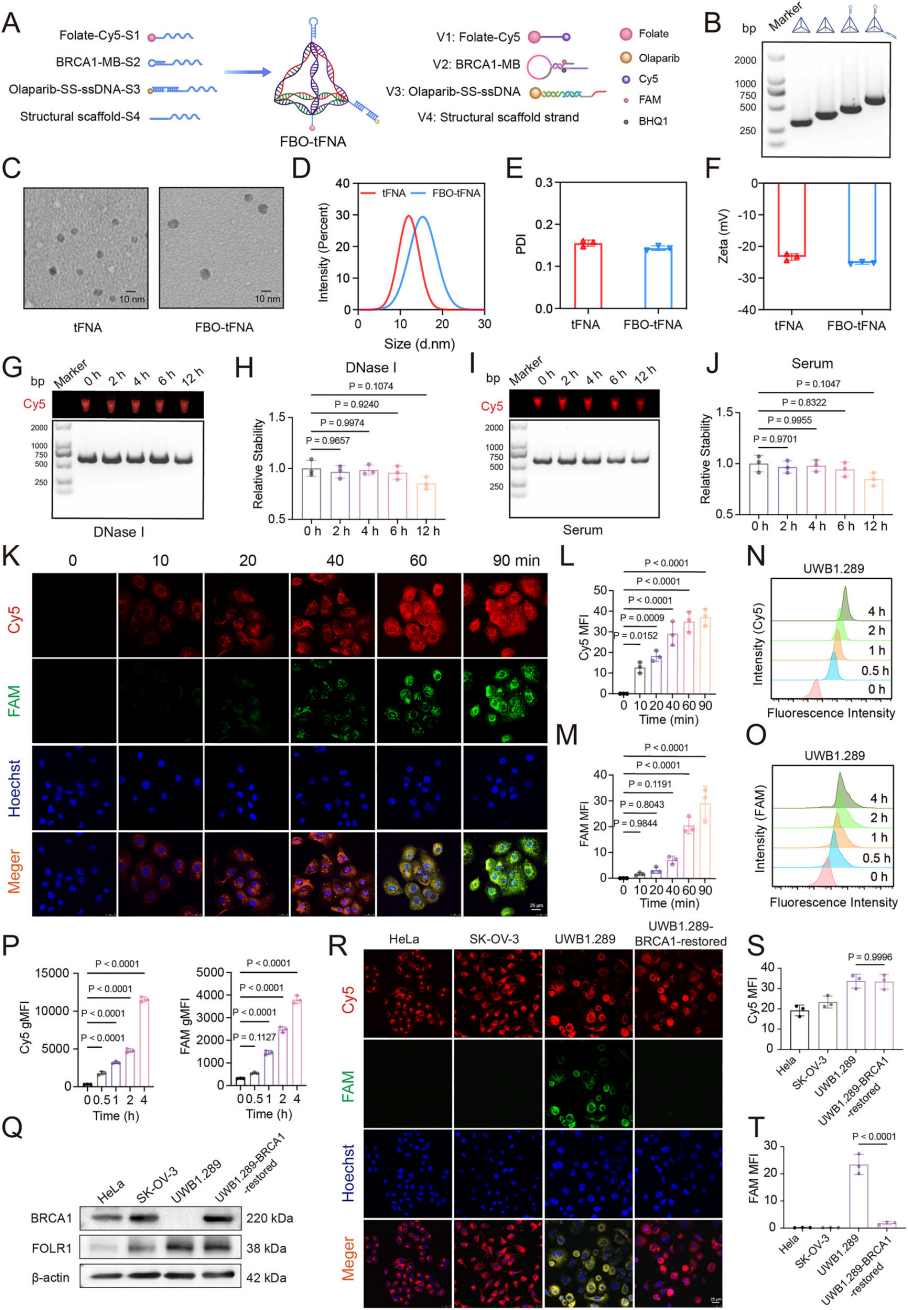
Rational design, controlled fabrication, stability, and BRCA1 mutant‐transcript recognition of FBO‐tFNA. (A) Schematic illustration of the modular design and stepwise assembly of FBO‐tFNA. V1: Folate‐Cy5 targeting module; V2: BRCA1 mutation‐transcript‐specific molecular beacon (BRCA1‐MB, FAM/BHQ1‐labeled); V3: Disulfide‐modified olaparib (Olaparib‐SS‐ssDNA); V4: structural scaffold vertex/strand. (B) Native PAGE analysis of the stepwise assembly of FBO‐tFNA. Lane samples: partial hybridization intermediates and intact FBO‐tFNA. (C) Representative transmission electron microscopy image of tFNA and FBO‐tFNA. Scale bar: 10 nm. (D–F) Physicochemical characterization of FBO‐tFNA. (D) Dynamic light scattering analysis of the hydrodynamic diameter distribution of tFNA and FBO‐tFNA. (E) Polydispersity index (PDI) of tFNA and FBO‐tFNA. (F) Zeta potential of tFNA and FBO‐tFNA. (G‐J) Structural stability of FBO‐tFNA under physiological conditions. (G) Native PAGE analysis of FBO‐tFNA co‐incubated with DNase I (10 U/mL) at 37°C for 0–12 h. (H) Quantification of band grayscale intensity from native PAGE analysis following DNase I treatment. (I) Native PAGE analysis of FBO‐tFNA incubated in 10% FBS at 37°C for 0–12 h. (J) Quantification of band grayscale intensity from native PAGE analysis following serum incubation. (K) Real‐time confocal laser scanning microscopy imaging of FBO‐tFNA internalization in UWB1.289 cells. Cy5 (folate targeting, red), FAM (BRCA1‐MB activation, green), and merged channels were acquired at indicated time points. Scale bar: 25 µm. (L,M) Quantification of Cy5 and FAM mean fluorescence intensities (MFI), respectively. (N,O) Representative flow‐cytometric fluorescence distributions of (N) Cy5 and (O) FAM in UWB1.289 cells at the indicated time points. (P) Quantification of Cy5 and FAM geometric mean fluorescence intensities (gMFI). (Q) Western blot analysis of BRCA1 and FOLR1 expression in HeLa, SK‐OV‐3, UWB1.289, and UWB1.289‐BRCA1‐restored cells. (R) Confocal imaging of FBO‐tFNA uptake and activation in the indicated cell lines. Cy5 (red), FAM (green), and merged channels. Scale bar: 25 µm. (S,T) Quantification of Cy5 and FAM mean fluorescence intensities in indicated cell lines. Statistical significance was evaluated using one‐way ANOVA (H, J, L, M, P). Statistical significance was evaluated using t‐test analysis (S, T).

Fluorescently labeled FBO‐tFNA was used to evaluate intracellular delivery and mutation‐responsive activation in UWB1.289 cells, whereas nonfluorescent FBO‐tFNA was used for therapeutic and mechanistic assays unless otherwise specified. Cy5 fluorescence was used as a surrogate indicator of nanoparticle‐associated cellular internalization, while FAM fluorescence reflected BRCA1 mutant‐transcript recognition and beacon activation. Confocal imaging revealed an “uptake‐before‐activation” pattern: Cy5 accumulated at the cell membrane within about 10 min and was internalized by approximately 40 min, whereas cytoplasmic FAM fluorescence increased at about 90 min and colocalized with Cy5 (Figure 1K–M). Flow cytometry similarly showed that Cy5‐positive cells reached a plateau at around 1 h, while FAM intensity continued to rise (Figure 1N–P), consistent with folate receptor‐mediated uptake followed by BRCA1 mutant RNA‐triggered toehold strand displacement. Flow cytometry further characterized surface FOLR1 expression across HeLa, SK‐OV‐3, UWB1.289, and UWB1.289‐BRCA1‐restored cells. Cy5‐labeled FBOA‐tFNA uptake varied among these cell models, and competition with excess free folic acid reduced Cy5 fluorescence in UWB1.289 cells, supporting the contribution of folate receptor‐mediated cellular internalization (Figure S6A–D). Selectivity was further examined in cells with different FR‐α expression levels and BRCA1 statuses (Figure 1Q and Figure S5I). Cy5 fluorescence showed an association with FR‐α expression and was used as an indicator of nanoparticle‐associated uptake, whereas robust FAM activation occurred only in BRCA1 2594delC‐mutant UWB1.289 cells and remained near background in SK‐OV‐3 and UWB1.289‐BRCA1‐restored cells, consistent with BRCA1 mutant‐transcript recognition‐dependent beacon activation (Figure 1R–T) [35, 37, 38]. Together, these findings support two complementary layers of selectivity involving folate receptor‐mediated cellular internalization and BRCA1 mutant‐transcript‐responsive molecular recognition, providing a basis for evaluating mutation‐selective PARP inhibition, DNA damage accumulation, and synthetic lethality.

To distinguish FR‐α‐mediated uptake from BRCA1 mutant‐transcript recognition, we compared FBO‐tFNA with a scrambled‐beacon control (F‐scrMB‐O‐tFNA). Cy5 fluorescence was comparable between the two constructs in both UWB1.289 and UWB1.289‐BRCA1‐restored cells, whereas strong FAM activation was observed only in BRCA1‐mutant UWB1.289 cells treated with FBO‐tFNA (Figure S6E–G). The scrambled‐beacon control also showed weaker cytotoxicity than FBO‐tFNA in UWB1.289 cells (Figure S6H). These results indicate that FR‐α primarily mediates cellular uptake, while the BRCA1‐responsive molecular beacon provides an additional intracellular genotype‐recognition layer. Importantly, beacon activation serves as a readout of sequence‐specific BRCA1 mutant‐transcript recognition rather than directly triggering drug release, which is independently mediated by reduction of the disulfide linker. Together, these findings support FR‐α‐mediated targeting combined with BRCA1 mutation‐responsive intracellular recognition.

### 2.2 BRCA1 Mutation‐Associated Cytotoxicity and DNA Damage Response Induced by FBO‐tFNA

The mutation‐selective therapeutic rationale of FBO‐tFNA is illustrated in Figure 2A. FBO‐tFNA exerted marked concentration‐ and time‐dependent cytotoxicity in UWB1.289 cells, with an IC_50_ comparable to that of free olaparib (1.485 µM vs. 1.363 µM). In contrast, its toxicity was substantially attenuated in UWB1.289‐BRCA1‐restored cells and remained close to that of blank tFNA (Figure 2B,C). Annexin V/PI flow cytometry further showed that FBO‐tFNA induced ∼36% apoptosis in UWB1.289 cells, exceeding the ∼29% observed with olaparib, whereas FBO‐tFNA induced only approximately 2% apoptosis in BRCA1‐restored cells (Figure 2D,E). Consistently, colony formation assays demonstrated that FBO‐tFNA markedly impaired the long‐term proliferative capacity of UWB1.289 cells, while exerting limited effects on UWB1.289‐BRCA1‐restored cells (Figure 2F,G). These results indicate that the selective cytotoxicity of FBO‐tFNA is associated with BRCA1 mutation‐responsive intracellular recognition, consistent with the synthetic lethal vulnerability of BRCA‐deficient cells to PARP inhibition [39, 40].

**FIGURE 2:**
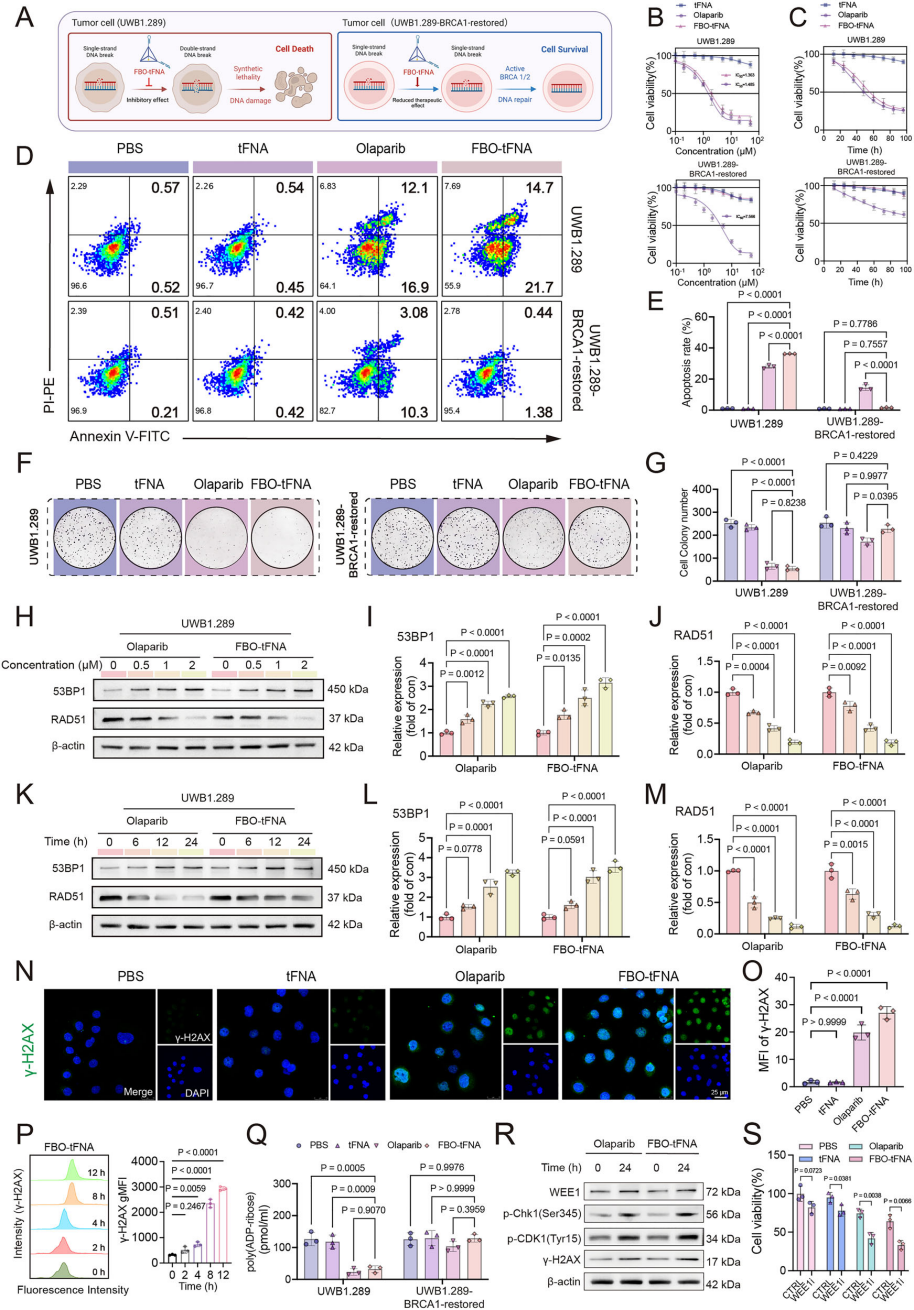
Mutation‐specific antitumor effects and DNA damage response of FBO‐tFNA in BRCA1‐mutant ovarian cancer. (A) Schematic illustration of the FBO‐tFNA mechanism of action. (B,C) Cell viability of UWB1.289 and UWB1.289‐BRCA1‐restored cells treated with PBS, tFNA, olaparib, and FBO‐tFNA at indicated concentrations (B) and time points (C). (D) Representative Annexin V‐FITC/PI staining flow cytometry plots of UWB1.289 and UWB1.289‐BRCA1‐restored cells after the indicated treatments. (E) Quantification of the apoptotic rate in each treatment group. (F) Colony formation assays of UWB1.289 and UWB1.289‐BRCA1‐restored cells after indicated treatments. (G) Quantification of colony number. (H‐J) Western blot analysis (H) and quantification of 53BP1 (I) and RAD51 (J) protein expression in UWB1.289 cells treated with FBO‐tFNA at indicated concentrations. (K–M) Western blot analysis (K) and quantification of 53BP1 (L) and RAD51 (M) protein expression in UWB1.289 cells treated with FBO‐tFNA at the indicated time points. (N) Representative immunofluorescence images of γ‐H2AX expression in UWB1.289 cells after different treatments. Green: γ‐H2AX; Blue: DAPI. Scale bar: 25 µm. (O) Quantification of γ‐H2AX mean fluorescence intensity (MFI). (P) Representative flow‐cytometric fluorescence distributions and quantitative analysis of γ‐H2AX geometric mean fluorescence intensity (gMFI) over time following FBO‐tFNA treatment. (Q) ELISA detection of poly(ADP‐ribose) levels in UWB1.289 and UWB1.289‐BRCA1‐restored cells. (R) Western blot analysis of WEE1, p‐Chk1(Ser345), p‐CDK1(Tyr15), and γ‐H2AX protein expression in UWB1.289 cells. (S) Evaluation of the synergistic cytotoxicity derived from the combination of a WEE1 inhibitor (WEE1i) with various formulation cohorts against UWB1.289 cells. Statistical significance was evaluated using one‐way ANOVA (E, G, I, J, L, M, O, P, Q, S).

Mechanistically, FBO‐tFNA induced a pronounced imbalance in DNA damage repair in UWB1.289 cells. Western blot analysis showed that increasing concentrations or prolonged exposure to FBO‐tFNA progressively increased 53BP1 expression while continuously decreasing RAD51 expression (Figure 2H–M), indicating persistent DNA damage accumulation accompanied by altered DNA‐damage repair signaling and reduced HR‐associated repair capacity [40]. γ‐H2AX immunofluorescence further revealed that FBO‐tFNA induced substantially more DNA damage foci than free olaparib, while flow cytometry confirmed progressive accumulation of γ‐H2AX signals within 0–12 h of treatment (Figure 2N–P). In addition, ELISA showed that FBO‐tFNA significantly reduced intracellular poly(ADP‐ribose) levels in UWB1.289 cells, whereas only limited changes were observed in BRCA1‐restored cells (Figure 2Q), suggesting preferential PARP inhibition in the BRCA1‐mutant cellular context.

Further analysis showed that FBO‐tFNA treatment simultaneously increased WEE1, p‐Chk1(Ser345), p‐CDK1(Tyr15), and γ‐H2AX levels in UWB1.289 cells (Figure 2R and Figure S7A–D). In particular, increased p‐CDK1(Tyr15) reflected compensatory activation of the G2/M checkpoint in response to sustained DNA damage, suggesting that tumor cells may depend on WEE1‐mediated checkpoint protection to delay damage‐induced death. Accordingly, combination treatment with a WEE1 inhibitor further enhanced the cytotoxic effect of FBO‐tFNA (Figure 2S). Bliss independence analysis first confirmed intrinsic pharmacological synergy between free olaparib and adavosertib across multiple dose combinations (Figure S9A). Importantly, positive Bliss interactions were also retained when PARP inhibition was delivered through FBO‐tFNA in combination with adavosertib, with the strongest synergy observed around the intermediate IC_50_‐based dose range (Figure S9B). Previous studies have likewise demonstrated that WEE1 inhibition weakens checkpoint protection in DNA‐damaged cells and potentiates olaparib‐mediated cytotoxicity [16, 41, 42]. Thus, FBO‐tFNA produces BRCA1 mutation‐associated PARP inhibition, but also induces persistent DNA damage and checkpoint dependence, providing both mechanistic and quantitative support for incorporating WEE1 inhibition into the subsequent dual‐drug FBOA‐tFNA platform.

### 2.3 FBOA‐tFNA‐Mediated Spatiotemporal Coordination of PARPi/WEE1i Efficiently Kills BRCA1‐Mutant Tumor Cells

The above results showed that FBO‐tFNA induced sustained DNA damage in BRCA1‐mutant cells together with G2/M checkpoint activation. To overcome this protective response, we introduced a disulfide‐linked WEE1 inhibitor module, Adavosertib‐SS‐ssDNA, at the fourth tFNA vertex, generating the dual‐drug nanoplatform FBOA‐tFNA for coordinated intracellular delivery of olaparib and adavosertib (Figure 3A and Figure S8A–J) [30, 41]. High‐performance liquid chromatography (HPLC) analysis further showed that the loading efficiency of adavosertib in FBOA‐tFNA was approximately 78%, confirming efficient incorporation of the WEE1 inhibitor module into the dual‐drug nanoplatform (Figure S5D‐G,H). At the standard 1.0‐equivalent input, the experimentally achieved drug‐to‐tFNA ratios were approximately 0.80 for olaparib and 0.79 for adavosertib, indicating a near‐equimolar dual‐drug composition of the assembled FBOA‐tFNA (Figure S5G,H). To further distinguish free drug species from linker‐associated forms, LC–MS/MS analysis was performed. Representative chromatograms showed that olaparib and adavosertib were predominantly present as linker‐bound species under non‐reducing conditions, whereas GSH treatment increased the corresponding free drug signals (Figure S5J). Quantification based on LC–MS/MS peak‐area integration further demonstrated a shift from linker‐bound to free drug fractions following GSH exposure (Figure S5K), confirming reduction‐responsive disulfide cleavage‐mediated drug liberation. Intracellular HPLC analysis further showed that free Ola+Ada exhibited progressive divergence of the intracellular Ola/Ada ratio from ∼1.3 at 1 h to ∼3.0 at 8 h, whereas FBOA‐tFNA maintained a ratio close to 1:1 throughout the 1–8 h period (Figure S9C–E), supporting more coordinated intracellular drug‐associated exposure. Further analysis showed that olaparib‐associated intracellular levels declined more slowly after FBO‐tFNA treatment than after free olaparib, indicating a more sustained intracellular exposure profile over 48 h (Figure S9F). In vitro release analysis further showed that 10 mM GSH markedly accelerated olaparib and adavosertib liberation from the cleavable constructs, reaching approximately 83% and 77% at 24 h, respectively, whereas release remained low from the corresponding non‐cleavable constructs under the same conditions (Figure S9G,H). These results support a disulfide‐dependent, reduction‐responsive drug‐liberation mechanism. Intracellular glutathione analysis showed that the reduced glutathione (GSH)/oxidized glutathione (GSSG) ratio decreased from ∼145 in the PBS group to ∼58 following FBOA‐tFNA treatment, indicating a treatment‐associated shift toward a more oxidized intracellular redox state (Figure 3B). L‐buthionine‐(S, R)‐sulfoximine (BSO) treatment alone had little effect on cell viability, whereas BSO pretreatment partially attenuated FBOA‐tFNA‐induced cytotoxicity, increasing cell viability from ∼40% to 65% (Figure 3C). These findings are consistent with a contribution of intracellular GSH to the reduction‐responsive activity of FBOA‐tFNA. In addition to human BRCA1 2594delC‐mutant UWB1.289 cells, we further established a stable mouse ID8 cell model carrying the BRCA1 truncation mutation 972delG (ID8‐BRCA1‐mut) to evaluate the applicability of this strategy across different BRCA1‐mutant backgrounds (Figure S8K). Cell Counting Kit‐8 (CCK‐8) assays showed that the IC_50_ values of FBOA‐tFNA in UWB1.289 and ID8‐BRCA1‐mut cells were 0.821 and 0.621 µM, respectively, markedly lower than those of FBO‐tFNA (1.469 and 1.736 µM) and comparable to those of free Ola+Ada (0.724 and 0.746 µM) (Figure 3D). Annexin V/PI flow cytometry further showed that FBOA‐tFNA induced apoptosis rates of ∼70% and ∼66% in the two mutant cell lines, respectively, which were significantly higher than those induced by FBO‐tFNA (∼35%) and comparable to the free Ola+Ada group (Figure 3E,F). These results indicate that FBOA‐tFNA effectively enables PARPi/WEE1i combinational killing in BRCA1‐mutant cells, consistent with the mechanistic basis that BRCA‐deficient cells are sensitive to PARP inhibition and that WEE1 blockade enhances PARPi efficacy [39, 42, 43].

**FIGURE 3:**
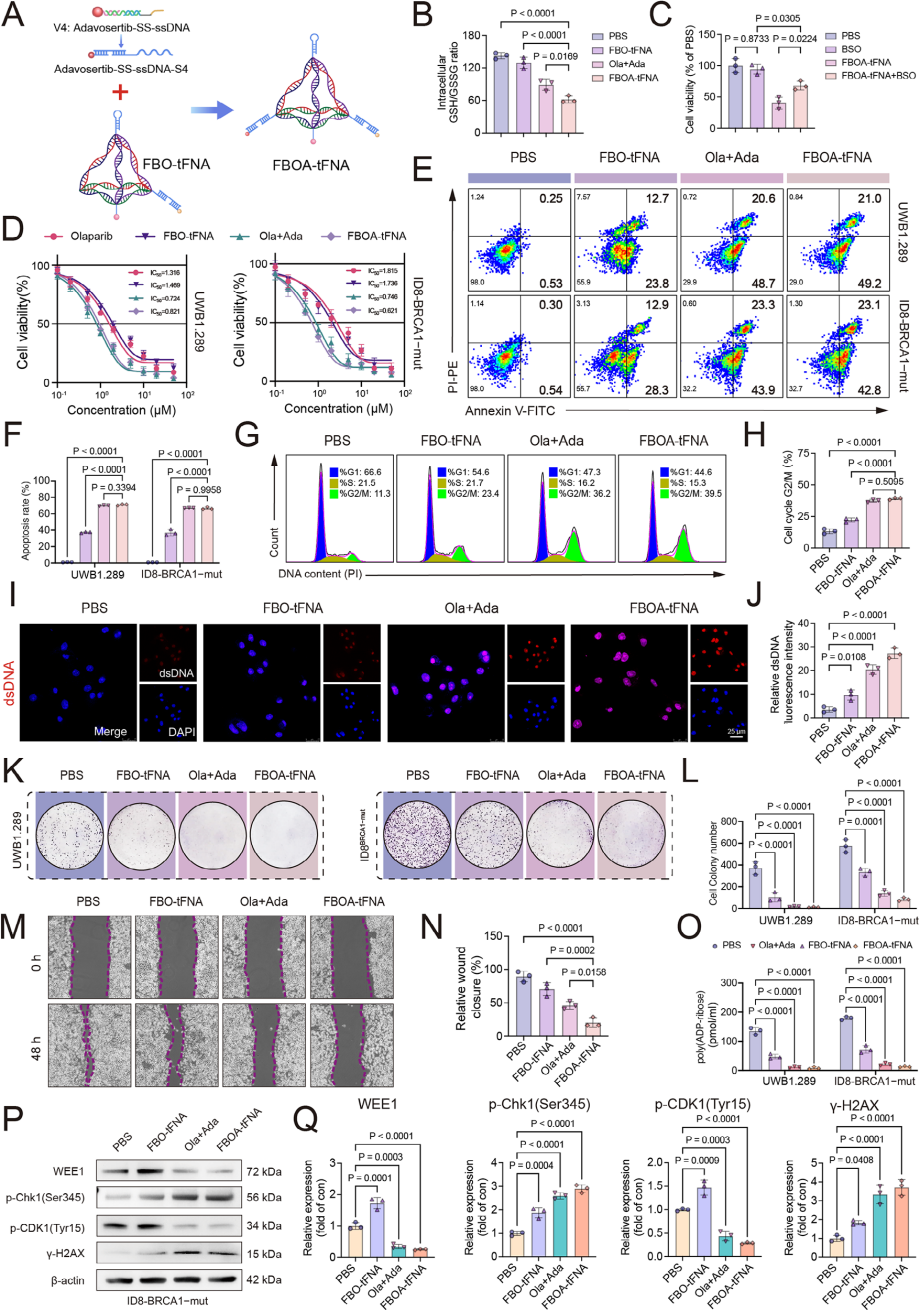
Synergistic killing of BRCA1‐mutant ovarian cancer by FBOA‐tFNA through PARPi/WEE1i dual‐drug combination. (A) Schematic illustration of FBOA‐tFNA construction. Adavosertib‐SS was integrated at the V4 vertex of FBO‐tFNA to construct the PARPi/WEE1i dual‐drug synergistic system. (B) Intracellular GSH/GSSG ratios in UWB1.289 cells following the indicated treatments. (C) Cell viability of UWB1.289 cells following the indicated BSO and FBOA‐tFNA treatments. (D) Cell viability of UWB1.289 and ID8‐BRCA1‐mut cells treated with olaparib, FBO‐tFNA, Ola+Ada, and FBOA‐tFNA for 48 h. (E) Representative Annexin V‐FITC/PI staining flow cytometry plots of UWB1.289 and ID8‐BRCA1‐mut cells after the indicated treatments. (F) Quantification of apoptotic rate in each treatment group. (G) Cell cycle analysis of ID8‐BRCA1‐mut cells after the indicated treatments. (H) Quantification of the G2/M‐phase cell population in each treatment group. (I) Representative immunofluorescence images of intracellular dsDNA signals in ID8‐BRCA1‐mut cells after different treatments. Red: dsDNA; Blue: DAPI. Scale bar: 25 µm. (J) Quantification of intracellular dsDNA mean fluorescence intensity (MFI). (K) Colony formation assays of UWB1.289 and ID8‐BRCA1‐mut cells after the indicated treatments. (L) Quantification of colony number. (M) Representative images of scratch wound healing assays in ID8‐BRCA1‐mut cells after the indicated treatments. (N) Quantification of relative migration rate. (O) ELISA detection of poly(ADP‐ribose) levels in UWB1.289 and ID8‐BRCA1‐mut cells across treatment groups. (P) Western blot analysis of WEE1, p‐Chk1(Ser345), p‐CDK1(Tyr15), and γ‐H2AX protein expression in ID8‐BRCA1‐mut cells after different treatments. (Q) Quantification of relative protein expression levels. Statistical significance was evaluated using one‐way ANOVA (B, C, F, H, J, L, N, O, Q).

Cell‐cycle analysis showed that FBOA‐tFNA and free Ola+Ada disrupted G2/M checkpoint regulation more strongly than FBO‐tFNA (Figure 3G,H). FBOA‐tFNA also increased intracellular dsDNA signals (Figure 3I,J), consistent with sustained DNA damage following mutation‐responsive intracellular recognition and reduction‐responsive drug liberation. Colony formation and wound‐healing assays further showed marked inhibition of clonogenic growth and wound closure (Figure 3K–N), indicating that incorporation of the WEE1 inhibitory module converted FBO‐tFNA‐induced DNA damage into stronger cell‐cycle disruption and tumor‐cell killing [16, 42]. Enzyme‐linked immunosorbent assay (ELISA) further showed that FBOA‐tFNA reduced intracellular poly(ADP‐ribose) levels more strongly than FBO‐tFNA (Figure 3O). Western blotting revealed decreased WEE1 and p‐CDK1(Tyr15), together with increased p‐Chk1(Ser345) and γ‐H2AX levels (Figure 3P,Q). These changes indicate attenuation of WEE1‐mediated G2/M checkpoint protection and persistence of DNA damage signaling. FBOA‐tFNA therefore promoted aberrant mitotic entry before complete DNA repair, consistent with combined PARP/WEE1 inhibition [16, 41, 42, 43]. Together with the Bliss analysis and intracellular HPLC results, these findings indicate that the enhanced therapeutic effect reflects both the intrinsic pharmacological synergy between PARP and WEE1 inhibition and the improved temporal coordination of intracellular olaparib‐ and adavosertib‐associated exposure achieved by FBOA‐tFNA. This coordinated combination further strengthens checkpoint abrogation, persistent DNA damage, and tumor‐cell killing.

### 2.4 Patient‐Derived Ovarian Cancer Organoids Expressing a BRCA1^2594delC^‐Containing Transcript Validate the Mutation‐Responsive Recognition and Therapeutic Potential of FBOA‐tFNA

To further enhance the clinical relevance of our evaluation, patient‐derived ovarian cancer organoids were established to assess the mutation‐responsive activation and therapeutic efficacy of FBOA‐tFNA in a physiologically relevant ex vivo model. An engineered partial BRCA1 construct containing the 2594delC‐associated mutant sequence was introduced into ovarian cancer organoids to generate BRCA1^2594delC^‐containing transcript‐expressing organoids, with empty vector‐transduced organoids serving as controls (Figure 4A). The construct contained an N‐terminal 4 × FLAG tag and encoded a predicted 348‐amino‐acid product of approximately 39.1 kDa (Figure S10). Western blot analysis confirmed FLAG‐tagged construct expression only in the transcript‐expressing organoids, whereas no detectable signal was observed in the empty vector (EV) group. High folate receptor expression was also detected in both organoid models, supporting the feasibility of folate‐mediated FBOA‐tFNA uptake (Figure 4B). We next examined whether FBOA‐tFNA retained its mutation‐responsive recognition behavior in organoids. Confocal imaging showed efficient Cy5 fluorescence in both EV and BRCA1^2594delC^‐containing transcript‐expressing organoids, indicating robust nanoplatform uptake, whereas strong FAM activation was observed predominantly in transcript‐expressing organoids (Figure 4C). Quantification of fluorescence intensities confirmed comparable Cy5 uptake but markedly higher FAM activation in BRCA1^2594delC^‐containing transcript‐expressing organoids than in EV controls (Figure 4D). Consistently, flow cytometric analysis further showed efficient Cy5 accumulation in both groups, while FAM fluorescence was selectively enhanced in BRCA1^2594delC^‐containing transcript‐expressing organoids after FBOA‐tFNA incubation (Figure 4E). These results demonstrate that FBOA‐tFNA maintains efficient organoid uptake and sequence‐specific BRCA1 mutant‐transcript recognition in the patient‐derived organoid system.

**FIGURE 4:**
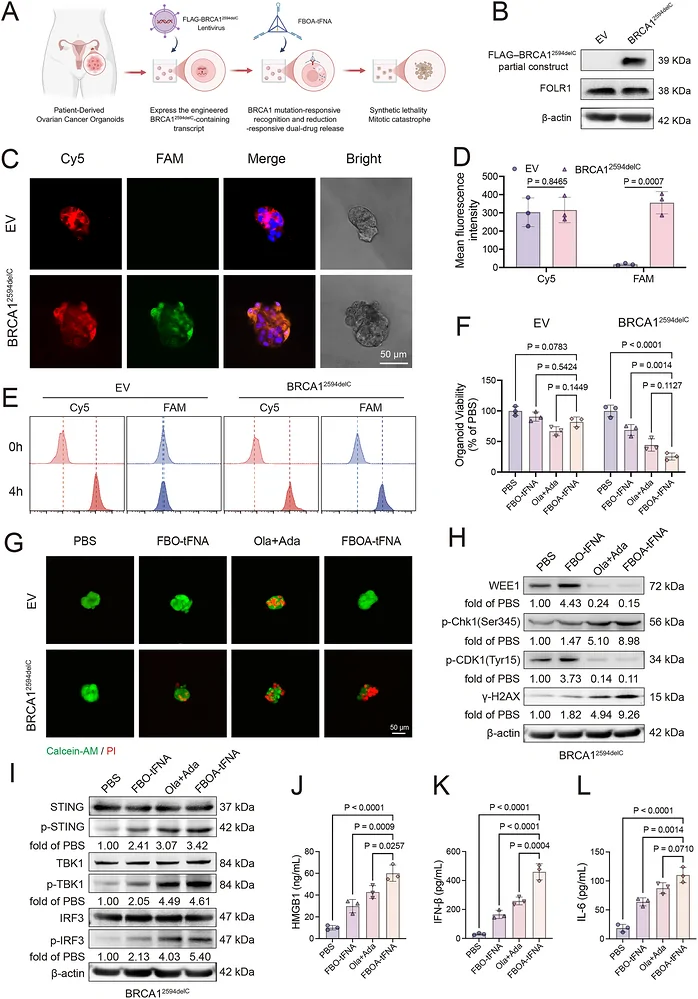
Mutation‐responsive recognition and therapeutic effects of FBOA‐tFNA in patient‐derived ovarian cancer organoids expressing a BRCA1^2594delC^‐containing transcript. (A) Schematic illustration of the establishment of patient‐derived ovarian cancer organoids, lentiviral transduction of an engineered partial BRCA1 construct containing the 2594delC‐associated mutant sequence, and FBOA‐tFNA‐mediated mutation‐responsive recognition and therapeutic response. (B) Western blot analysis of FLAG‐tagged engineered partial BRCA1 construct and FOLR1 expression in empty vector (EV) control and BRCA1^2594delC^‐containing transcript‐expressing organoids. β‐actin was used as the loading control. (C) Representative confocal fluorescence images of EV control and BRCA1^2594delC^‐containing transcript‐expressing organoids after incubation with FBOA‐tFNA. Cy5 fluorescence indicates FBOA‐tFNA uptake, FAM fluorescence indicates BRCA1 mutant‐transcript‐responsive molecular beacon activation, and Hoechst stains nuclei. Scale bar: 50 µm. (D) Quantification of Cy5 and FAM mean fluorescence intensities in EV control and BRCA1^2594delC^‐containing transcript‐expressing organoids shown in Figure 4C. (E) Flow cytometric analysis of Cy5 and FAM fluorescence in EV control and BRCA1^2594delC^‐containing transcript‐expressing organoids before and after FBOA‐tFNA incubation, showing efficient nanoplatform uptake and mutation‐responsive FAM activation. (F) ATP‐based viability analysis of EV control and BRCA1^2594delC^‐containing transcript‐expressing organoids after treatment with PBS, FBO‐tFNA, Ola+Ada, or FBOA‐tFNA. (G) Representative live/dead staining images of EV control and BRCA1^2594delC^‐containing transcript‐expressing organoids after treatment with PBS, FBO‐tFNA, Ola+Ada, or FBOA‐tFNA. Scale bar: 50 µm. (H) Western blot analysis of WEE1, p‐Chk1(Ser345), p‐CDK1(Tyr15), and γ‐H2AX expression in organoids after the indicated treatments. β‐actin was used as the loading control. (I) Western blot analysis of total and phosphorylated STING, TBK1, and IRF3 in organoids after the indicated treatments. Phosphorylation levels were quantified as p‐STING/STING, p‐TBK1/TBK1, and p‐IRF3/IRF3 ratios and expressed relative to the PBS group. β‐actin was used as the loading control. (J‐L) ELISA analysis of HMGB1 (J), IFN‐β (K), and IL‐6 (L) release in organoid culture supernatants after the indicated treatments. Statistical significance was evaluated using t‐test analysis (D). Statistical significance was evaluated using one‐way ANOVA (F, J, K, L).

Functional validation further demonstrated the superior antitumor activity of FBOA‐tFNA in BRCA1^2594delC^‐containing transcript‐expressing ovarian cancer organoids. Adenosine triphosphate (ATP)‐based viability analysis showed that FBOA‐tFNA exerted the strongest inhibitory effect on organoid viability, particularly in transcript‐expressing organoids, whereas EV control organoids showed a relatively weaker viability reduction under the same treatment conditions (Figure 4F). Live/dead staining consistently revealed pronounced organoid cell death after Ola+Ada or FBOA‐tFNA treatment, with the most evident cytotoxicity observed in the FBOA‐tFNA group, while EV organoids displayed less extensive dead‐cell staining than transcript‐expressing organoids (Figure 4G). Western blot analysis showed that FBOA‐tFNA decreased WEE1 and p‐CDK1 levels while increasing p‐Chk1 and γ‐H2AX expression, consistent with enhanced DNA damage and attenuation of G2/M checkpoint protection (Figure 4H). Moreover, FBOA‐tFNA increased p‐STING/STING, p‐TANK‐binding kinase 1 (TBK1) /TBK1, and p‐interferon regulatory factor 3 (IRF3)/IRF3 ratios and promoted the release of HMGB1, IFN‐β, and IL‐6 (Figure 4I–L). Together, these results demonstrate BRCA1 mutant‐transcript‐responsive molecular recognition by FBOA‐tFNA together with enhanced PARP/WEE1 inhibition‐associated DNA damage and STING–TBK1–IRF3 phosphorylation in patient‐derived ovarian cancer organoids, supporting its potential as an ex vivo mutation‐responsive therapeutic platform.

### 2.5 In Vivo Spatiotemporal Distribution and Repeated‐Dose Tolerability of FBOA‐tFNA

Given the favorable structural stability, biocompatibility, and in vivo delivery potential of tFNA [24, 29, 44], as well as the frequent overexpression of folate receptor in ovarian cancer and its utility as a tumor‐targeting delivery site [35, 38, 45], we further evaluated the in vivo biodistribution, BRCA1 mutation‐responsive molecular‐beacon activation, and repeated‐dose tolerability of FBOA‐tFNA. For in vivo targeting, biodistribution, and mutation‐responsive activation imaging, fluorescently labeled FBOA‐tFNA was used to enable Cy5‐based tracking of tumor accumulation and FAM‐based monitoring of molecular beacon activation. First, a UWB1.289 subcutaneous xenograft model was established, and the dynamic changes in Cy5 and FAM fluorescence signals were monitored using small‐animal in vivo fluorescence imaging. The results showed that both the Cy5 signal, which reflects tumor accumulation of the nanoplatform, and the FAM signal, which indicates BRCA1 mutation‐responsive molecular‐beacon activation, gradually increased over time, reached a peak at approximately 8 h after administration, and then progressively declined (Figure 5A–D). Ex vivo organ imaging further confirmed that the Cy5 signal was predominantly distributed in tumor tissues, with only weak fluorescence observed in the liver and kidney. In contrast, the FAM signal was mainly activated at the tumor site (Figure 5E,F). Single‐cell flow cytometric analysis of tumor tissues further confirmed increased Cy5 and FAM fluorescence intensities in the functionalized nanoplatform groups (Figure 5G,H).

**FIGURE 5:**
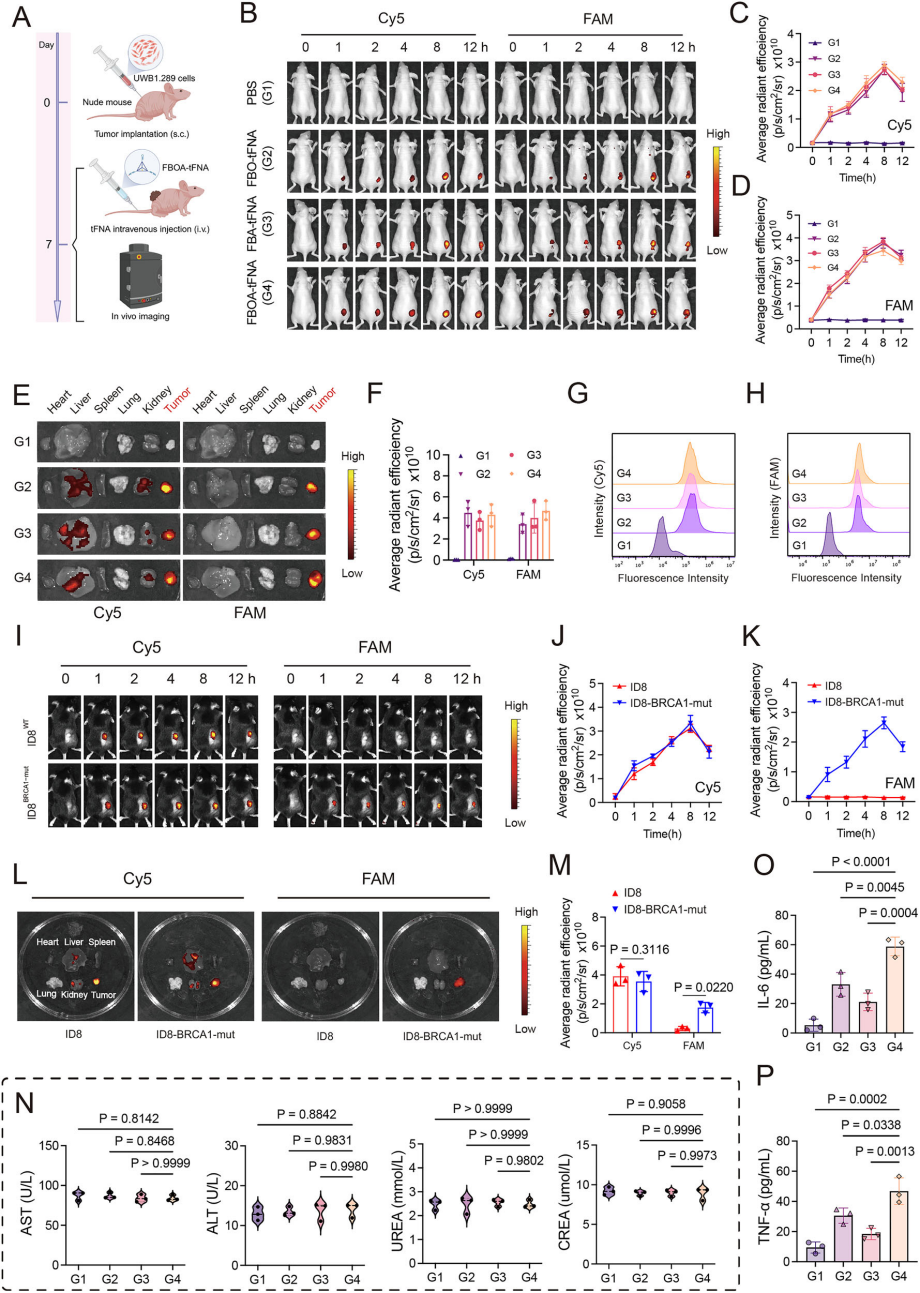
In vivo targeting distribution and repeated‐dose tolerability evaluation of FBOA‐tFNA. (A) Schematic illustration of the administration regimen in the UWB1.289 subcutaneous xenograft nude mouse model. G1: PBS; G2: FBO‐tFNA; G3: FBA‐tFNA; G4: FBOA‐tFNA. (B) In vivo imaging of mice at different time points after tail vein injection of the indicated formulations. Dynamic monitoring of Cy5 (nanoplatform tracking signal) and FAM (molecular beacon activation signal) at 0–12 h. (C,D) Quantification of average radiant efficiency. (C) Cy5 signal; (D) FAM signal. (E) Ex vivo fluorescence imaging of major organs and tumors at 12 h post‐administration. (F) Quantification of average radiant efficiency in ex vivo organs. (G–H) Flow cytometric analysis of Cy5 (G) and FAM (H) fluorescence in single‐cell suspensions prepared from tumor tissues at 12 h post‐administration. (I) In vivo imaging of mice at different time points after administration in a symmetrical subcutaneous tumor model of ID8 and ID8‐BRCA1‐mut. (J‐K) Quantification of average radiant efficiency. (J) Cy5 signal; (K) FAM signal. (L) Ex vivo fluorescence imaging of major organs and tumors at 12 h post‐administration. (M) Quantification of average radiant efficiency in ex vivo organs. (N) Serum biochemical parameters (AST, ALT, UREA, CREA) after five doses administered every 3 days. (O‐P) ELISA analysis of serum IL‐6 (O) and TNF‐α (P) levels after the indicated treatments. Statistical significance was evaluated using one‐way ANOVA (N‐P).

To further verify the dependence of FAM fluorescence on BRCA1 mutation status, we established a bilateral subcutaneous tumor model bearing ID8 and ID8‐BRCA1‐mut tumors. After intravenous administration of fluorescently labeled FBOA‐tFNA, both Cy5 and FAM signals were synchronously enhanced in ID8‐BRCA1‐mut tumors and similarly reached a peak at approximately 8 h post‐injection. In contrast, although Cy5‐mediated targeting and accumulation could be detected in ID8 tumors, the FAM signal remained close to the background level (Figure 5I–K). Ex vivo imaging further showed that Cy5 accumulation was comparable between the two tumor types, whereas markedly enhanced FAM fluorescence was observed only in BRCA1‐mutant tumors (Figure 5L,M). These results demonstrate that FBOA‐tFNA exhibits a well‐defined spatiotemporal behavior in vivo, characterized by time‐dependent tumor accumulation and BRCA1 mutation‐responsive molecular‐beacon activation, enabling mutation‐associated intracellular recognition at the tumor site.

Repeated‐dose tolerability evaluation showed that FBOA‐tFNA did not induce overt organ toxicity under the tested regimen. Serum biochemical analysis showed that liver and kidney function markers and tissue injury‐related indicators showed no appreciable abnormalities compared with those in the PBS group (Figure 5N and Figures S11A, S12A). H&E staining of major organs, including the heart, liver, spleen, lung, and kidney, showed no obvious inflammatory cell infiltration, tissue necrosis, or structural damage (Figures S11B and S12B). Notably, serum IL‐6 and TNF‐α levels were significantly elevated following FBOA‐tFNA treatment compared with the PBS group (Figure 5O,P), indicating a measurable systemic cytokine response under the tested dosing conditions [46, 47]. Taken together, these results indicate that FBOA‐tFNA enables efficient tumor‐targeted accumulation and BRCA1 mutation‐responsive molecular‐beacon activation in vivo while providing preliminary evidence of repeated‐dose tolerability at the biochemical and histopathological levels, although the observed cytokine elevation indicates that systemic immune activation should be considered in further safety evaluation.

### 2.6 FBOA‐tFNA Alters the Transcriptomic Landscape of BRCA1‐Mutant Ovarian Cancer Cells

To investigate the molecular effects of FBOA‐tFNA, RNA sequencing was performed on PBS‐ and FBOA‐tFNA‐treated cells (Figure 6A). Differential expression analysis identified 3091 genes, including 1266 upregulated and 1825 downregulated genes (Figure 6B). Hierarchical clustering clearly separated the two groups and showed good consistency among biological replicates, indicating extensive transcriptomic changes after treatment (Figure 6C). Gene Ontology (GO) and Kyoto Encyclopedia of Genes and Genomes (KEGG) analyses of the upregulated genes revealed enrichment in cellular and metabolic processes, membrane‐associated structures, lysosome, glycosaminoglycan degradation, glutathione metabolism, and autophagy‐related pathways (Figure 6D,E and Figure S13A). Gene set enrichment analysis (GSEA) further demonstrated positive enrichment of lysosome, lysosome organization, and autophagy gene sets (Figure 6G and Figure S14A,B). These changes are consistent with folate receptor‐mediated uptake followed by endosomal/lysosomal trafficking, intracellular processing, and reduction‐responsive drug release. They also suggest that the lysosome‐autophagy system may contribute to nanocarrier processing and adaptation to treatment‐induced cellular stress.

**FIGURE 6:**
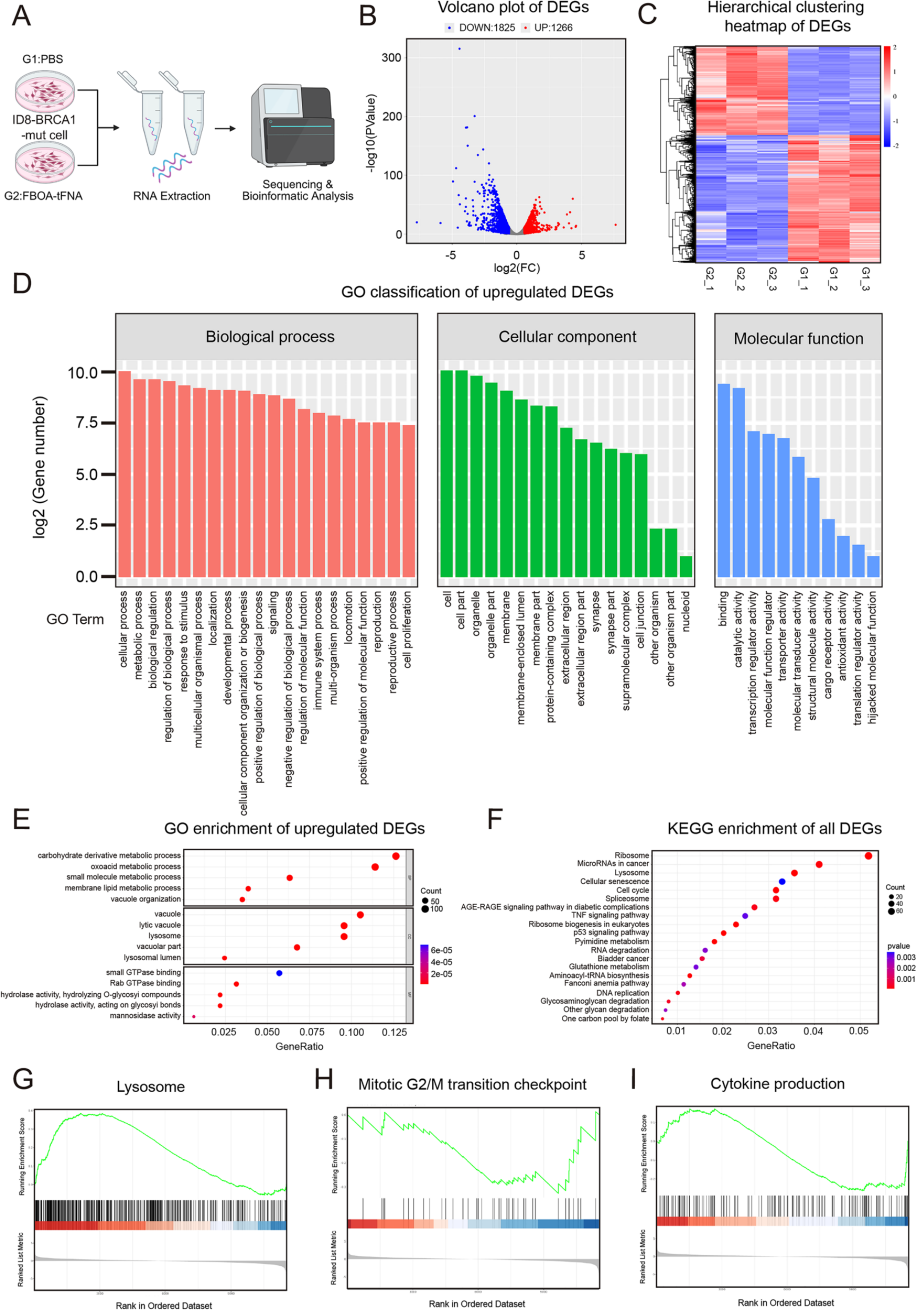
FBOA‐tFNA remodels the transcriptomic landscape of BRCA1‐mutant ovarian cancer cells. (A) Schematic illustration of RNA sequencing workflow for PBS‐ and FBOA‐tFNA‐treated cells. (B) Volcano plot of differentially expressed genes after FBOA‐tFNA treatment. Red indicates upregulated genes and blue indicates downregulated genes. (C) Hierarchical clustering heatmap of differentially expressed genes between PBS‐ and FBOA‐tFNA‐treated groups. (D) GO functional classification analysis of upregulated differentially expressed genes after FBOA‐tFNA treatment. (E) GO enrichment bubble plot of upregulated differentially expressed genes after FBOA‐tFNA treatment. (F) KEGG pathway enrichment bubble plot of all differentially expressed genes. (G‐I) GSEA showing enrichment of gene sets related to lysosome (G), mitotic G2/M transition checkpoint (H), and cytokine production (I) after FBOA‐tFNA treatment.

Conversely, downregulated genes were enriched in pathways associated with proliferation and biosynthetic activity, including ribosome, ribosome biogenesis, spliceosome, cell cycle, p53 signaling, pyrimidine metabolism, DNA replication, and nucleotide metabolism (Figure S13B). GO analysis similarly showed suppression of RNA processing, ribonucleoprotein complex biogenesis, rRNA metabolism, and rRNA processing (Figure S13C,D), indicating broad disruption of the transcriptional and translational programs required for rapid tumor‐cell growth. GSEA revealed negative enrichment of the mitotic G2/M transition checkpoint and DNA replication checkpoint gene sets (Figure 6H and Figure S14C), suggesting attenuation of programs required for replication stress responses, checkpoint maintenance, and cell‐cycle progression. These findings are consistent with the observed increases in γ‐H2AX and 53BP1, cell‐cycle disruption, reduced clonogenicity, and impaired proliferation (Figures 2 and 3), and support the effects of coordinated PARP/WEE1 inhibition on DNA damage and mitotic control.

Analysis of all differentially expressed genes further highlighted pathways related to lysosomes, cellular senescence, cell cycle, TNF signaling, AGE‐RAGE signaling, p53 signaling, and microRNAs in cancer (Figure 6F). In addition, cytokine production and Toll‐like receptor signaling gene sets were positively enriched (Figure 6I and Figure S14D), suggesting activation of inflammatory and innate immune‐related transcriptional programs [19, 46, 47]. Collectively, FBOA‐tFNA suppressed DNA replication, cell‐cycle progression, RNA processing, and ribosome biogenesis while promoting lysosomal, autophagic, cytokine, and innate immune transcriptional programs. These transcriptomic changes are consistent with treatment‐associated changes involving intracellular processing, DNA damage amplification, proliferative program suppression, and immune activation.

### 2.7 FBOA‐tFNA Promotes ICD‐Associated Tumor‐Cell Stress and Antitumor Immune Activation Accompanied by STING–TBK1–IRF3 Signaling

Transcriptomic enrichment of TNF signaling and cellular senescence‐related programs prompted us to examine whether FBOA‐tFNA‐induced tumor‐cell damage could generate immunostimulatory signals. In ID8‐BRCA1‐mut cells, confocal imaging revealed altered intracellular HMGB1 distribution after treatment, with the cytoplasmic‐to‐nuclear HMGB1 fluorescence ratio following the order FBOA‐tFNA > Ola+Ada > FBO‐tFNA (Figure 7A,B). This shift in the relative intracellular distribution of HMGB1 was most pronounced following FBOA‐tFNA treatment. ELISA confirmed increased extracellular HMGB1, while PD‐L1 expression showed a similar trend (Figure 7C–F). qPCR further demonstrated upregulation of CXCL10, CCL5, IFN‐β, and PD‐L1, with overall expression levels higher than those induced by Ola+Ada (Figure 7E). Together, these findings indicate that combined PARP/WEE1 inhibition not only causes direct cytotoxicity but also induces an ICD‐associated phenotype characterized by altered HMGB1 distribution, extracellular HMGB1 accumulation, and type I interferon‐associated signaling [46, 48, 49]. Because extracellular HMGB1 alone cannot distinguish regulated DAMP release from passive release secondary to membrane disruption, these findings are not interpreted as definitive evidence of bona fide immunogenic cell death. We next evaluated the STING signaling axis, given that DNA damage, genomic instability, and micronucleus formation can expose cytosolic DNA and activate innate DNA sensing [50, 51, 52]. FBOA‐tFNA produced the strongest p‐STING signal by confocal imaging (Figure 7G,H) and increased p‐STING/STING, p‐TBK1/TBK1, and p‐IRF3/IRF3 ratios by Western blotting (Figure 7I), supporting enhanced phosphorylation of the STING‐TBK1‐IRF3 signaling cascade. These results suggest that nanocarrier‐mediated coordinated PARP/WEE1 delivery may enhance intracellular DNA damage and thereby promote STING‐associated immune signaling, consistent with previous reports on PARP inhibition and combined PARP/WEE1 inhibition [17, 49, 53]. Western blotting further showed increased cleaved caspase‐3/total caspase‐3 levels following combined PARP/WEE1 inhibition, supporting enhanced apoptosis execution (Figure 7J). Pharmacological STING inhibition attenuated both STING–TBK1–IRF3 phosphorylation and FBOA‐tFNA‐induced bone marrow‐derived dendritic cell (BMDC) maturation, supporting a substantial STING‐dependent component of this immunostimulatory response (Figure S15A‐C).

**FIGURE 7:**
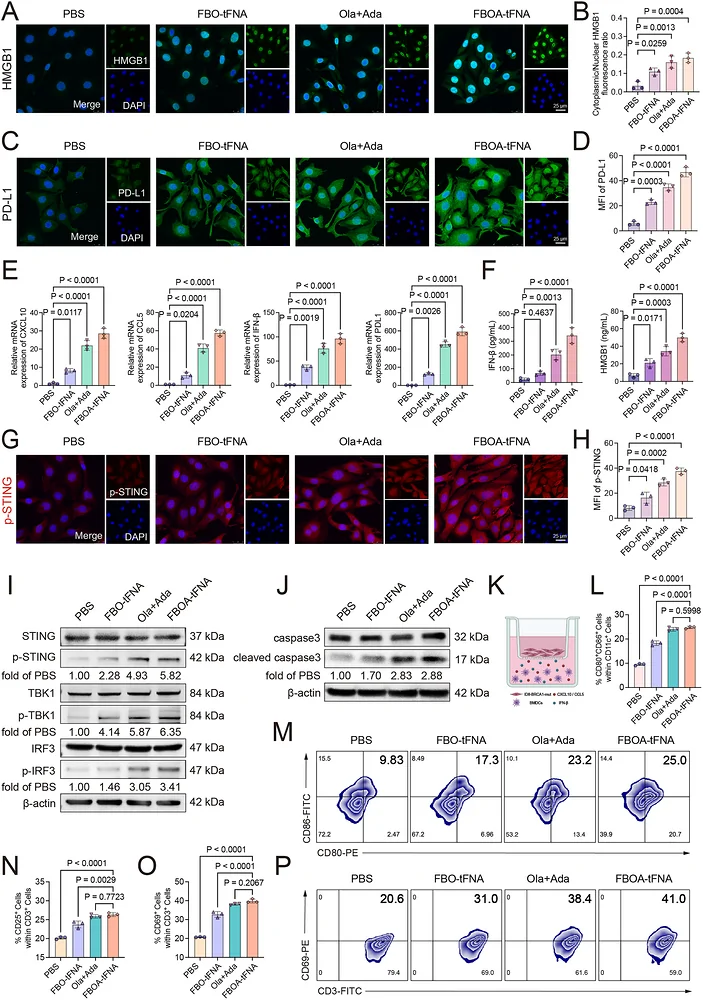
FBOA‐tFNA promotes ICD‐associated tumor‐cell stress and antitumor immune activation accompanied by STING–TBK1–IRF3 signaling. (A) Representative immunofluorescence images showing HMGB1 expression and altered intracellular distribution in ID8‐BRCA1‐mut cells after the indicated treatments. Green: HMGB1; Blue: DAPI. Scale bar: 25 µm. (B) Quantification of the cytoplasmic‐to‐nuclear HMGB1 fluorescence ratio. (C) Representative immunofluorescence images of PD‐L1 expression in ID8‐BRCA1‐mut cells after the indicated treatments. Green: PD‐L1; Blue: DAPI. Scale bar: 25 µm. (D) Quantification of PD‐L1 MFI. (E) Relative mRNA expression of CXCL10, CCL5, IFN‐β, and PD‐L1 in each treatment group. (F) ELISA detection of IFN‐β secretion and HMGB1 release in each treatment group. (G) Representative immunofluorescence images of p‐STING expression in ID8‐BRCA1‐mut cells after indicated treatments. Red: p‐STING; Blue: DAPI. Scale bar: 25 µm. (H) Quantification of p‐STING MFI. (I) Western blot analysis of total and phosphorylated STING, TBK1, and IRF3 in ID8‐BRCA1‐mut cells after indicated treatments. Phosphorylation levels were quantified as p‐STING/STING, p‐TBK1/TBK1, and p‐IRF3/IRF3 ratios and expressed relative to the PBS group. β‐actin was used as the loading control. (J) Western blot analysis of total and cleaved caspase‐3 after the indicated treatments. The cleaved caspase‐3/total caspase‐3 ratio was expressed relative to the PBS group. (K) Schematic illustration of separate Transwell coculture assays used to evaluate BMDC maturation and T‐cell activation. For the T‐cell activation assay, T cells were cocultured with pretreated ID8‐BRCA1‐mut tumor cells in an independent Transwell setup, followed by flow cytometric analysis of CD25 and CD69 expression within CD3^+^ cells. (L) Quantification of CD80^+^CD86^+^ mature DC proportions. (M) Representative flow cytometry plots of CD80^+^CD86^+^ mature DC proportions induced by each treatment group. (N) Quantification of CD25^+^ T cell proportions within CD3^+^ cells in each treatment group. (O) Quantification of CD69^+^ T cell proportions within CD3^+^ cells in each treatment group. (P) Representative flow cytometry plots of CD69^+^ T cell proportions within CD3^+^ cells in each treatment group. Statistical significance was evaluated using one‐way ANOVA (B, D‐F, H, L, N, O).

Tumor cells pretreated with FBOA‐tFNA increased the proportion of CD80^+^CD86^+^ mature BMDCs to approximately 25%, compared with 23.2% for Ola+Ada and 17.3% for FBO‐tFNA (Figure 7K–M and Figure S16A). In the independent T‐cell Transwell assay, CD25^+^ and CD69^+^ T cells reached approximately 27% and 41%, respectively, approaching the Ola+Ada group and exceeding the FBO‐tFNA group (Figure 7N–P and Figure S16B,C). Collectively, FBOA‐tFNA was associated with enhanced dendritic‐cell maturation and T‐cell activation following DNA damage‐associated tumor‐cell stress, supporting coordinated innate and adaptive antitumor immune responses and subsequent combination with immune checkpoint blockade [46, 47, 48, 54].

### 2.8 FBOA‐tFNA Enhances In Vivo Antitumor Efficacy and Coordinated Antitumor Immune Responses in a Syngeneic Subcutaneous Ovarian Tumor Model

To evaluate the in vivo therapeutic efficacy of FBOA‐tFNA, a syngeneic ID8‐BRCA1‐mut subcutaneous ovarian tumor model was established, followed by five intravenous injections according to the treatment schedule (Figure 8A). FBOA‐tFNA markedly delayed tumor growth, as shown by endpoint tumor photographs and tumor growth curves (Figure 8B,C). At the endpoint, FBOA‐tFNA reduced tumor weight to 0.31 ± 0.05 g, corresponding to a ∼72.8% reduction compared with the PBS group (1.15 ± 0.09 g), and outperformed both FBO‐tFNA and FBA‐tFNA (Figure 8D). Body weight remained generally stable across all treatment groups, and no animal exhibited a body‐weight loss exceeding 15% relative to baseline (Figure 8E), indicating that FBOA‐tFNA enhanced PARP/WEE1 dual‐inhibition efficacy without obvious treatment‐associated body‐weight loss. Multiparameter flow cytometry further showed that FBOA‐tFNA not only suppressed tumor growth but was also associated with enhanced antitumor immune‐cell responses in subcutaneous tumors. Compared with the PBS group, FBOA‐tFNA increased the intratumoral proportion of CD3^+^CD4^+^ T cells from ∼35.8% to ∼44.2% and CD3^+^CD8^+^ T cells from ∼29.0% to ∼39.5% (Figure 8F,L,M and Figure S17A). In the spleen, CD8^+^CD69^+^ early activated T cells increased to ∼5.78%, approximately threefold higher than that in the PBS group (Figure 8G,N and Figure S17B). Moreover, the proportion of mature dendritic cells (CD80^+^CD86^+^/CD11c^+^) in tumors increased from ∼18.7% to ∼31%, NK cell infiltration increased from ∼5.17% to ∼18.6%, and CD8^+^IFN‐γ^+^ effector T cells in draining lymph nodes reached ∼12.3%, approximately eightfold higher than that in the PBS group (Figure 8H–J,O–Q and Figure S17C–E). These findings indicate that FBOA‐tFNA treatment was accompanied by enhanced dendritic‐cell maturation, T‐cell responses, and NK‐cell infiltration, supporting coordinated innate and adaptive antitumor immune responses [17, 46, 47, 50, 55].

**FIGURE 8:**
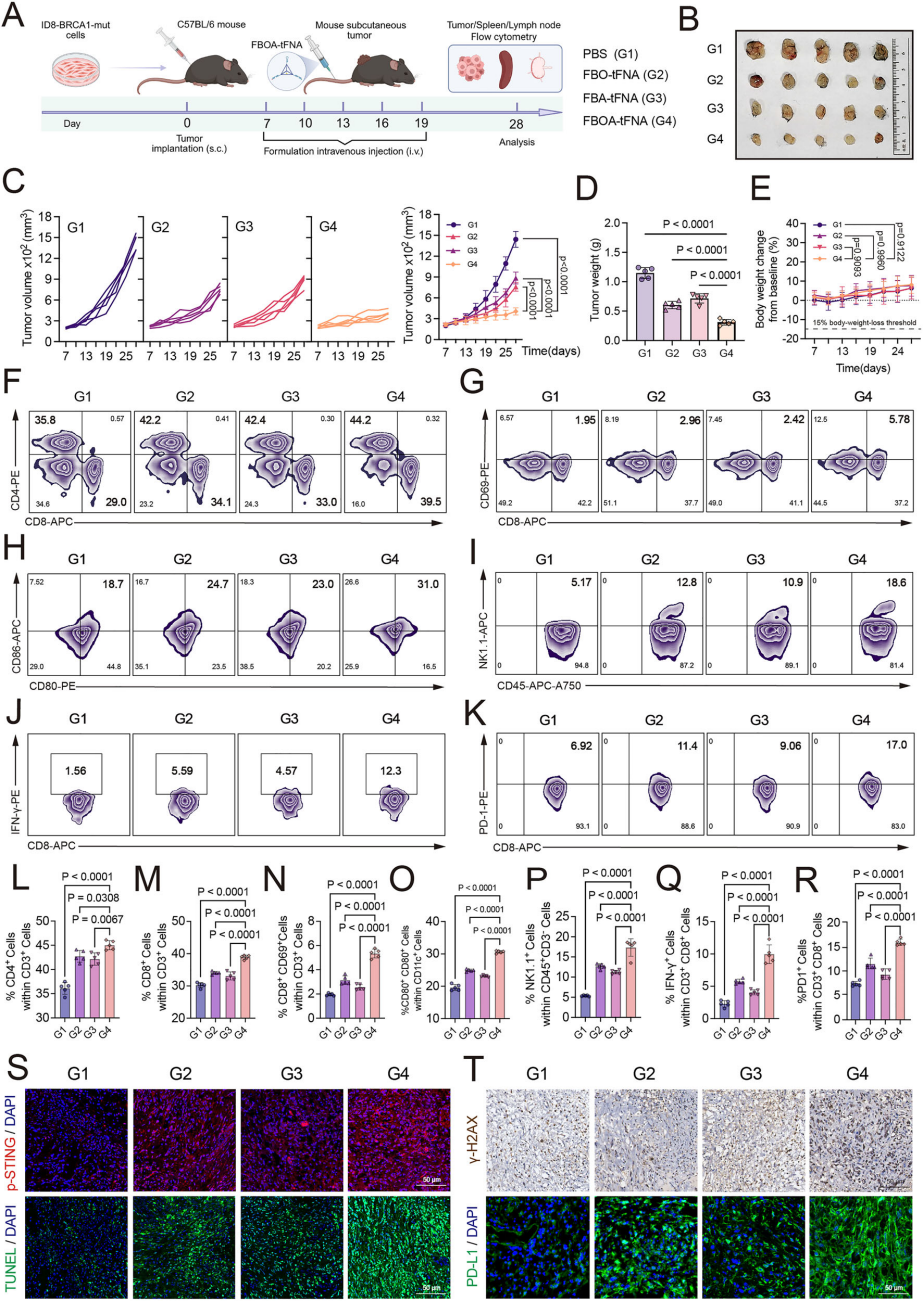
Antitumor efficacy and coordinated antitumor immune responses of FBOA‐tFNA in a BRCA1‐mutant syngeneic subcutaneous ovarian tumor model. (A) Schematic illustration of the administration regimen in the ID8‐BRCA1‐mut syngeneic subcutaneous ovarian tumor model. G1: PBS; G2: FBO‐tFNA; G3: FBA‐tFNA; G4: FBOA‐tFNA. (B) Photographs of tumors at the endpoint. (C) Tumor growth curves. (D) Tumor weight at the endpoint. (E) Body weight is expressed as percentage change relative to the baseline value before treatment. The dashed line indicates the predefined 15% body‐weight‐loss threshold. (F) Flow cytometric analysis of tumor‐infiltrating T cells. (G) Expression of the T cell activation marker CD69 in splenic effector T cells. (H) Expression of dendritic cell maturation markers CD80/CD86 in tumor tissues. (I) Analysis of NK cell infiltration in tumor tissues. (J) Proportions of CD8^+^IFN‐γ^+^ effector T cells in tumor‐draining lymph nodes. (K) Proportions of PD‐1^+^CD8^+^ T cells in tumor tissues. (L‐R) Quantification of immune cell populations. (L) CD4^+^ T cells; (M) CD8^+^ T cells; (N) CD8^+^CD69^+^ T cells; (O) CD11c^+^CD80^+^CD86^+^ dendritic cells; (P) CD45^+^CD3^−^NK1.1^+^ NK cells; (Q) CD8^+^IFN‐γ^+^ T cells; (R) PD‐1^+^CD8^+^ T cells. (S) Immunofluorescence staining of p‐STING/TUNEL in tumor tissues. Red: p‐STING; Green: TUNEL; Blue: DAPI. Scale bar: 50 µm. (T) Immunohistochemical staining of γ‐H2AX and immunofluorescence staining of PD‐L1 in tumor tissues. Scale bar: 50 µm. Statistical analysis was performed using two‐way ANOVA (C, E), and statistical significance was evaluated using one‐way ANOVA (D, L‐R).

Histological and multiplex immunofluorescence analyses further supported the tumor‐cell damage and immune‐associated changes described above. p‐STING and TUNEL staining showed enhanced STING phosphorylation and apoptotic signals in tumor tissues from the FBOA‐tFNA group, consistent with enhanced STING‐associated signaling and tumor‐cell apoptosis in vivo (Figure 8S and Figure S18A,B). γ‐H2AX/PD‐L1 staining revealed that tumors with stronger γ‐H2AX staining also showed increased PD‐L1 expression (Figure 8T and Figure S18C,D), suggesting that FBOA‐tFNA‐induced PARP/WEE1 combined DNA damage was associated with increased PD‐L1 expression and adaptive immune‐checkpoint feedback. Notably, PD‐1 expression on tumor‐infiltrating CD8^+^ T cells increased from ∼5.7% in the PBS group to ∼16.3% in the FBOA‐tFNA group (Figure 8K,R and Figure S17F), suggesting that FBOA‐tFNA enhances antitumor immune responses while being accompanied by increased PD‐1‐associated adaptive immune feedback. These results provide a mechanistic rationale for subsequent combination therapy with anti‐PD‐1 treatment.

### 2.9 FBOA‐tFNA Outperforms Free PARP/WEE1 Inhibition in Peritoneal Tumor Control and Antitumor Immune Activation

To evaluate FBOA‐tFNA in a model that more closely reflects advanced ovarian cancer, including peritoneal dissemination and malignant ascites, we established an ID8‐BRCA1‐mut ‐Luc intraperitoneal dissemination model (Figure 9A). Malignant ascites not only reflects tumor burden but also forms a fluid microenvironment that promotes peritoneal metastasis, therapeutic resistance, and immune escape [56, 57]. In vivo bioluminescence imaging showed rapid signal expansion in the PBS group, whereas free Ola+Ada markedly reduced tumor burden, with marked bioluminescence reduction observed in 2 of 5 mice. FBOA‐tFNA produced the strongest response, with intraperitoneal signals nearly disappearing in 3 of 5 mice (Figure 9B). These results indicate that nanoplatform‐mediated coordinated delivery of PARP and WEE1 inhibitors achieved better intraperitoneal tumor control than conventional free dual‐drug administration, consistent with the antitumor activity of combined PARP/WEE1 inhibition [17, 58].

**FIGURE 9:**
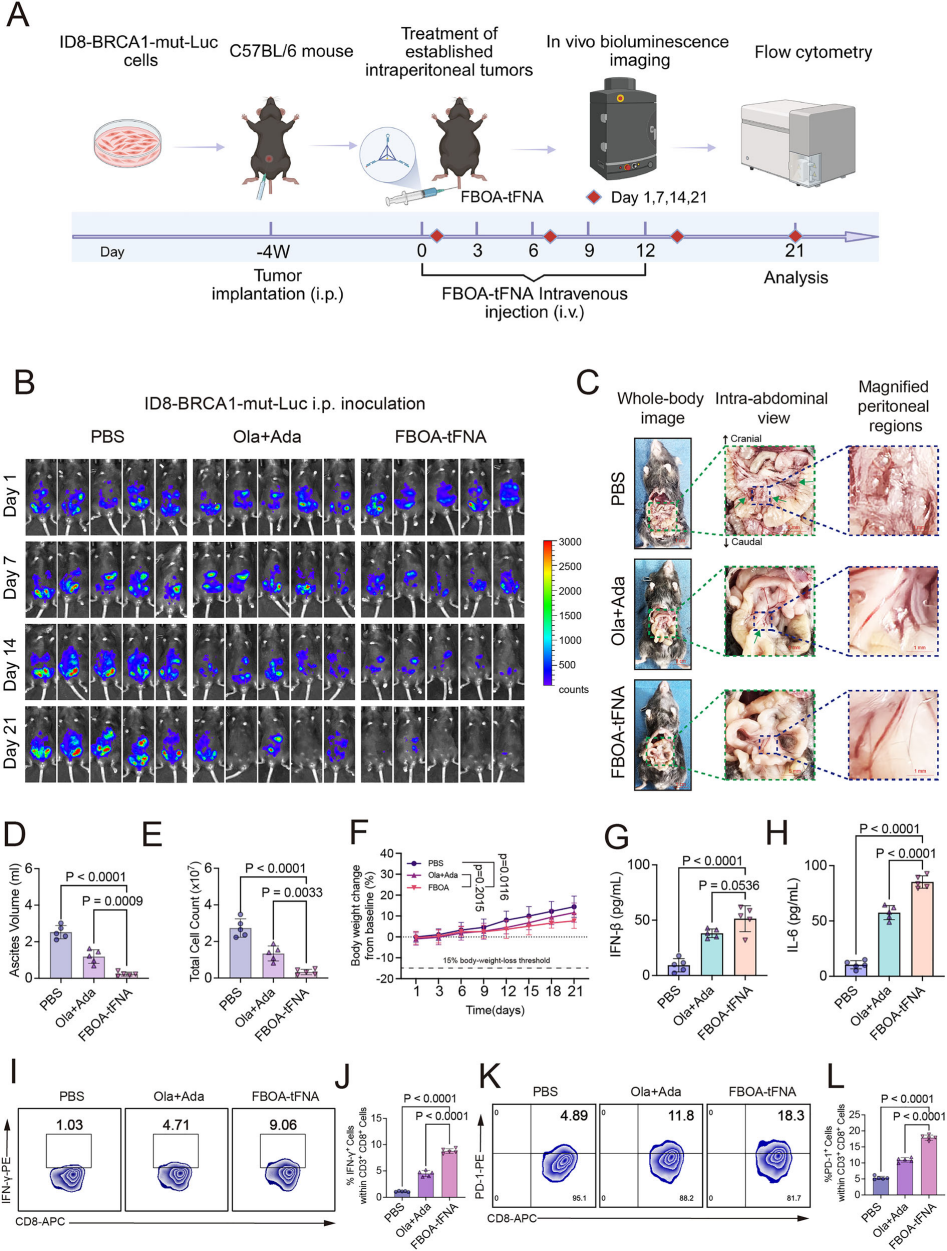
FBOA‐tFNA‐mediated peritoneal tumor control and antitumor immune responses in an intraperitoneal dissemination model. (A) Schematic illustration of the therapeutic regimen and multi‐dimensional evaluation in the intraperitoneal dissemination model of ID8‐BRCA1‐mut‐Luc cells. G1: PBS; G2: olaparib + adavosertib (Ola+Ada); G3: FBOA‐tFNA. (B) Dynamic monitoring of peritoneal tumor progression via in vivo bioluminescence imaging. (C) Representative images of intra‐abdominal tumor dissemination after different treatments. Whole‐body images, intra‐abdominal views, and magnified abdominal regions are presented for each treatment group. Green dashed boxes indicate the selected intra‐abdominal regions, and blue dashed boxes indicate the corresponding magnified regions. Green arrows indicate visible peritoneal metastatic nodules. Cranial and caudal anatomical orientations are indicated in the intra‐abdominal views. Scale bars are shown in each image. (D) Quantitative evaluation of terminal malignant ascites volume. (E) Total suspended cell count analysis in malignant ascites. (F) Body weight is expressed as percentage change relative to the baseline value before treatment. The dashed line indicates the predefined 15% body‐weight‐loss threshold. (G‐H) ELISA analysis of IFN‐β (G) and IL‐6 (H) levels in malignant ascites. (I‐J) Multicolor flow cytometric profiling (I) and statistical quantification (J) of CD8^+^IFN‐γ^+^ effector cytotoxic T lymphocytes (CTLs) in tumor‐draining lymph nodes (dLNs). (K‐L) Immunophenotypical analysis (K) and statistical quantification (L) of the compensatory adaptive PD‐1^+^ expression on the surface of malignant ascites‐infiltrating CD8^+^ T cells. Two‐way ANOVA was implemented for longitudinal statistical analysis (F), and one‐way ANOVA was utilized for terminal statistical significance evaluations (D, E, G, H, J, L).

Endpoint necropsy further confirmed suppression of peritoneal dissemination and ascites formation. Extensive metastatic nodules were present on the peritoneum, mesentery, and perivascular regions in PBS‐treated mice, whereas FBOA‐tFNA markedly reduced metastatic lesions and preserved a largely smooth peritoneal surface (Figure 9C). Ascites volume decreased to approximately 0.2 mL after FBOA‐tFNA treatment, compared with approximately 2.5 mL in the PBS group and 1.0 mL in the Ola+Ada group, with a corresponding reduction in total ascitic cell number (Figure 9D,E). Body weight remained generally stable throughout treatment, with no animal showing a decrease greater than 15% from baseline (Figure 9F). ELISA showed that FBOA‐tFNA increased ascitic IFN‐β to approximately 50 pg/mL and IL‐6 to approximately 85 pg/mL (Figure 9G,H), suggesting local type I interferon and inflammatory responses within the peritoneal cavity. Flow cytometry further showed enhanced antitumor immune responses. CD8^+^IFN‐γ^+^ effector T cells in tumor‐draining lymph nodes increased to 9.06% in the FBOA‐tFNA group, compared with 4.71% after Ola+Ada and 1.03% after PBS (Figure 9I,J and Figure S19A). Meanwhile, PD‐1 expression on ascites‐infiltrating CD8^+^ T cells increased to approximately 18.3%, compared with 4.89% in the PBS group (Figure 9K,L and Figure S19B). This finding suggests that FBOA‐tFNA promotes cytotoxic T‐cell responses while also inducing PD‐1‐associated adaptive immune feedback. Because the PD‐1/PD‐L1 axis can limit sustained CD8^+^ T‐cell activity [59, 60, 61], these results support combining FBOA‐tFNA with anti‐PD‐1 therapy. Collectively, FBOA‐tFNA suppressed peritoneal dissemination, reduced malignant ascites, and enhanced antitumor immune responses within the peritoneal tumor setting.

### 2.10 FBOA‐tFNA Enhances the Response to Anti‐PD‐1 Therapy, Accompanied by Enhanced Antitumor T‐Cell Responses and Memory‐Associated Phenotypes

Given the compensatory increase in PD‐1 on CD8^+^ T cells after FBOA‐tFNA (abbreviated as FBOA in this figure) treatment, we evaluated whether FBOA‐tFNA could sensitize tumors to anti‐PD‐1 antibody (αPD‐1) therapy in the ID8‐BRCA1‐mut‐Luc subcutaneous model (Figure 10A). FBOA‐tFNA was administered first, followed by αPD‐1 24 h later, to allow tumor‐cell damage and associated immune activation to precede checkpoint blockade. Longitudinal tumor measurements and bioluminescence imaging showed that the combination produced the strongest antitumor effect, with markedly flattened growth curves and near‐complete loss of bioluminescent signal. Mean tumor weight fell to 0.09 ± 0.04 g, representing a 92.2% reduction versus PBS (1.11 ± 0.06 g) and significantly lower values than FBOA‐tFNA alone (0.31 ± 0.05 g) or αPD‐1 alone (0.53 ± 0.08 g) (Figure 10B–E). Body weights remained generally stable during treatment, and no animal experienced a body‐weight loss exceeding the predefined 15% threshold (Figure 10F). These findings are consistent with reports that PARP inhibition enhances antitumor immunity through STING‐associated mechanisms and that PD‐1 blockade can further amplify this response [46, 49, 53, 62]. Tumor cytokine analysis showed that combination therapy increased IFN‐β and IL‐6 to approximately 65 and 95 pg/mL, respectively, above monotherapy and control levels (Figure 10G,H). Immunophenotyping further indicated changes in adaptive immune‐cell phenotypes. FBOA‐tFNA alone increased PD‐1^+^CD44^+^ exhausted‐like T cells to approximately 14.4%, whereas adding αPD‐1 reduced this population to approximately 0.53% (Figure 10I,L and Figure S20A). Meanwhile, PD‐1^−^CD44^+^ effector memory precursor cells increased to approximately 37%, compared with 29.6% after FBOA‐tFNA and 30.5% after αPD‐1 (Figure 10I,M). These results suggest that FBOA‐tFNA treatment followed by PD‐1 blockade is associated with reduced exhausted‐like T‐cell proportions and increased memory‐associated T‐cell populations [46, 49, 62, 63].

**FIGURE 10:**
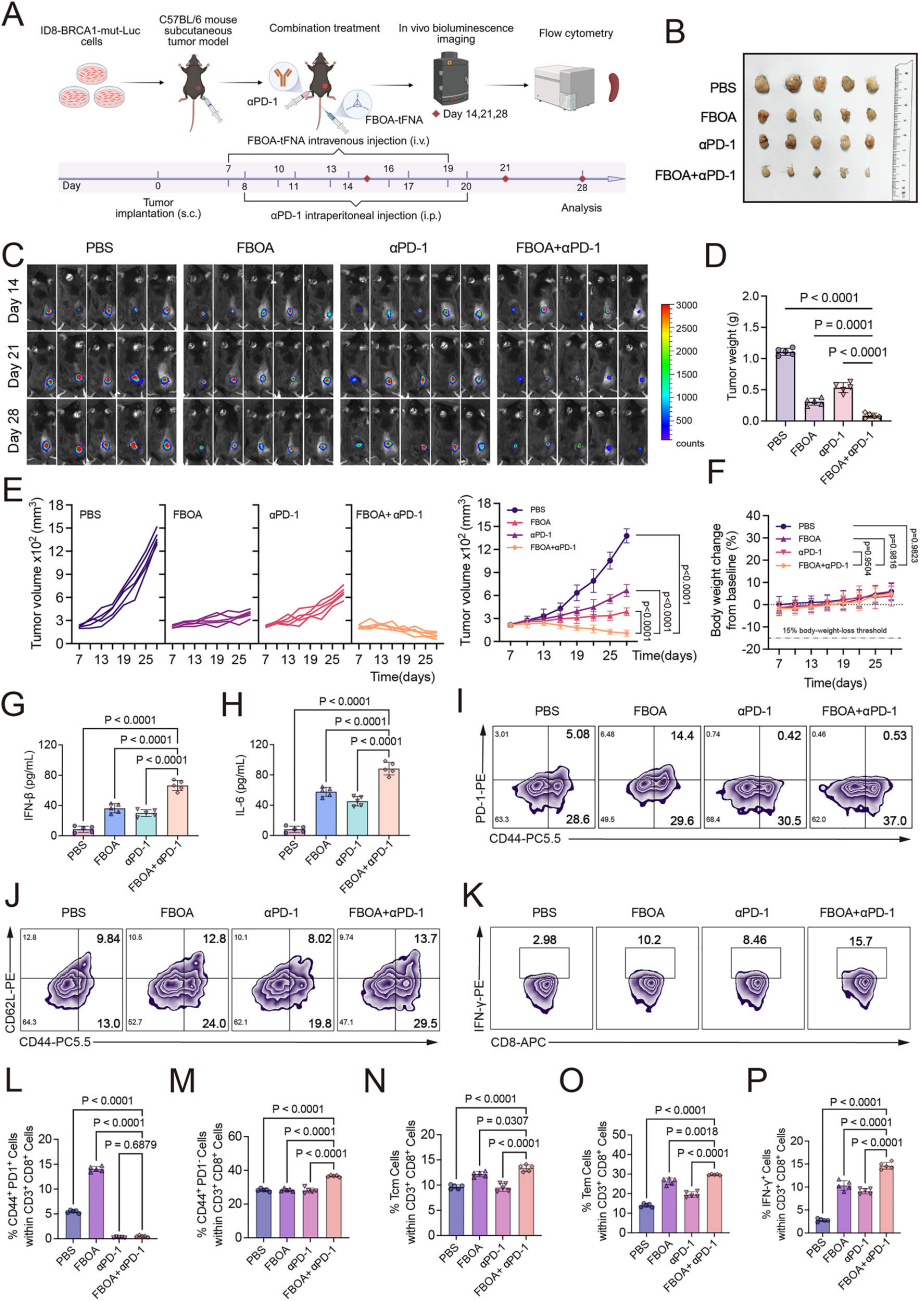
FBOA‐tFNA enhances the response to anti‐PD‐1 therapy, accompanied by reduced exhausted‐like T‐cell populations and enhanced memory‐associated T‐cell phenotypes. (A) Schematic illustration of the administration regimen in the ID8‐BRCA1‐mut‐Luc subcutaneous tumor model combined with anti‐PD‐1 antibody. (B) Photographs of tumors at the endpoint. (C) Longitudinal in vivo bioluminescence imaging of tumor progression. (D) Tumor weight at the endpoint. (E) Tumor growth curves. (F) Body weight is expressed as percentage change relative to the baseline value before treatment. The dashed line indicates the predefined 15% body‐weight‐loss threshold. (G,H) ELISA analysis of IFN‐β (G) and IL‐6 (H) in tumor tissues. (I) Flow cytometric analysis of exhausted‐like T cells in tumor tissues. (J) Flow cytometric analysis of central memory T cells (Tcm: CD44^+^CD62L^+^) and effector memory T cells (Tem: CD44^+^CD62L^−^) in the spleen. (K) Flow cytometric analysis of CD8^+^IFN‐γ^+^ effector T cells in tumor‐draining lymph nodes. (L–P) Quantification of immune cell populations. (L) CD44^+^PD‐1^+^ exhausted T cells; (M) CD44^+^PD‐1^−^ effector memory precursor cells; (N) Tcm cells; (O) Tem cells; (P) CD8^+^IFN‐γ^+^ T cells. Statistical analysis was performed using two‐way ANOVA (E,F), and statistical significance was evaluated using one‐way ANOVA (D,G,H, L‐P).

Memory‐related immune responses were then assessed in distal lymphoid organs. In the spleen, central memory T cells (Tcm; CD44^+^CD62L^+^) and effector memory T cells (Tem; CD44^+^CD62L^−^) reached approximately 13.7% and 29.5%, respectively, in the combination group, exceeding both monotherapies (Figure 10J,N,O and Figure S20B). CD8^+^IFN‐γ^+^ effector T cells in tumor‐draining lymph nodes also increased to approximately 15.7%, compared with 10.2% after FBOA‐tFNA and 8.46% after αPD‐1 (Figure 10K,P and Figure S20C). Collectively, sequential FBOA‐tFNA and αPD‐1 treatment strengthened tumor control and was accompanied by enhanced effector T‐cell responses and memory‐associated T‐cell phenotypes. This strategy supports further evaluation of nano‐immunotherapeutic combinations for BRCA1‐mutant ovarian cancer.

## 3 Discussion

In this study, we developed a BRCA1 mutation‐responsive tetrahedral framework nucleic acid nanoplatform, termed FBOA‐tFNA, which integrates mutant‐transcript recognition with coordinated PARP/WEE1 dual‐drug delivery. Through folate receptor‐mediated uptake, BRCA1 mutant RNA‐responsive molecular beacon activation, and reduction‐responsive intracellular drug release, this system improves the coordination of PARP and WEE1 inhibition in BRCA1‐mutant ovarian cancer models. Compared with conventional free olaparib plus adavosertib administration, FBOA‐tFNA offers several advantages, including tumor‐targeted accumulation, mutant‐transcript‐responsive beacon activation, coordinated intracellular dual‐drug delivery, enhanced antitumor efficacy, and preliminary repeated‐dose tolerability at the biochemical and histopathological levels under the tested regimens.

Mechanistically, FBOA‐tFNA amplifies DNA damage, weakens WEE1‐mediated G2/M checkpoint protection, induces cell‐cycle dysregulation and mitotic catastrophe, and promotes tumor‐cell death. This damage cascade is accompanied by STING‐associated signaling, type I interferon responses, chemokine secretion, ICD‐associated tumor‐cell stress, dendritic‐cell maturation, CD8^+^ T‐cell responses, and NK‐cell infiltration, together with enhanced antitumor immunity. These findings are consistent with recent studies highlighting the therapeutic relevance of STING pathway modulation in cancer treatment and immune activation [64]. Patient‐derived ovarian cancer organoids expressing an engineered partial BRCA1 construct containing the 2594delC‐associated mutant sequence further supported the mutation‐responsive recognition and therapeutic activity of FBOA‐tFNA in an ex vivo expression model. In vivo, FBOA‐tFNA exhibited efficient tumor accumulation, mutant‐transcript‐responsive beacon activation, potent antitumor efficacy, preliminary repeated‐dose tolerability despite a measurable systemic cytokine response, enhanced antitumor immune responses, an enhanced therapeutic response to anti‐PD‐1 blockade, and increased memory‐associated T‐cell phenotypes. Overall, this study establishes a programmable mutation‐responsive nano‐immunotherapeutic strategy that combines sequence‐specific molecular recognition with coordinated dual‐drug delivery and reduction‐responsive intracellular drug release.

Several limitations of the present study should be acknowledged. First, although FBOA‐tFNA showed enhanced antitumor activity in BRCA1‐mutant models, the current study was not designed to evaluate acquired PARP inhibitor‐resistant disease or long‐term resistance evolution, which will require dedicated resistant models and longitudinal studies. Second, although dendritic‐cell, CD8^+^ T‐cell, and NK‐cell responses were increased following treatment, immune‐cell depletion experiments were not performed; therefore, the indispensable contributions of individual immune‐cell populations to tumor control remain to be established. In addition, immunosuppressive populations such as regulatory T cells and myeloid‐derived suppressor cells were not comprehensively characterized. Third, although altered intracellular HMGB1 distribution and extracellular HMGB1 accumulation were consistent with ICD‐associated tumor‐cell stress, the absence of additional canonical ICD markers, including surface calreticulin and extracellular ATP, precludes definitive classification of bona fide immunogenic cell death. Finally, the present repeated‐dose safety evaluation did not assess anti‐tFNA/anti‐scaffold antibody generation or long‐term immunogenicity. Future studies incorporating resistant tumor models, broader immune profiling, immune‐cell depletion, comprehensive ICD assessment, and extended immunogenicity evaluation will be important to further define the therapeutic mechanism, durability, and translational potential of FBOA‐tFNA.

## 4 Experimental Section/Methods

### 4.1 Cell Culture

Human ovarian cancer UWB1.289 cells (CRL‐2945C, RRID: CVCL_B079) were purchased from Shanghai Yaji Biotechnology Co., Ltd. (Shanghai, China). UWB1.289‐BRCA1‐restored cells (SC7614, RRID: CVCL_B078) and murine ovarian cancer ID8 cells (SC6886, RRID: CVCL_IU14) were obtained from Shanghai Shuocheng Bio Co., Ltd. (Shanghai, China). SK‐OV‐3 cells (HTB‐77, RRID: CVCL_0532) were purchased from Haixing Bio (Suzhou, China), and HeLa cells (CCL‐2, RRID: CVCL_0030) were obtained from the American Type Culture Collection (ATCC). ID8‐BRCA1‐mut cells stably expressing truncated BRCA1(972delG) were generated by lentiviral transduction of parental ID8 cells with a BRCA1(972delG)‐encoding vector (PR‐004210, Zaiji Biotech), followed by selection with puromycin (2 µg mL^−1^; Invitrogen, ant‐pr‐1). ID8‐BRCA1‐mut‐Luc cells were generated by transducing ID8‐BRCA1‐mut cells with a firefly luciferase lentiviral vector and selecting with hygromycin B (100 µg mL^−1^; Invitrogen, ant‐hm‐1). All cell lines were authenticated by short tandem repeat (STR) profiling. UWB1.289 and UWB1.289‐BRCA1‐restored cells were cultured in UWB1.289‐specific medium (Shanghai Chengsuo, SC7614‐WP), whereas other cell lines were maintained in DMEM (Shanghai Life‐iLab Biotech Co., Ltd, AC01L043) supplemented with 10% fetal bovine serum (FBS; FBS‐BE500, NEWZERUM, Ltd.). Cells were cultured at 37°C in a humidified incubator with 5% CO_2_, and morphology was routinely monitored to ensure culture consistency. All cell lines were routinely tested and confirmed negative for mycoplasma contamination.

### 4.2 Patient‐Derived Ovarian Cancer Organoid Culture and Lentiviral Infection

Patient‐derived ovarian cancer organoids were cultured according to the manufacturer's protocol. The use of patient‐derived ovarian cancer organoids was approved by the Ethics Committee of Fujian Maternity and Child Health Hospital (Approval No. 2026KY166‐01), and written informed consent was obtained before sample collection. Briefly, organoids were embedded in organoid matrix gel and cultured in ovarian cancer organoid medium A or B (Daxiang Technology, OC100145/OC100146). The medium was changed every 3–4 days, and organoid growth was monitored regularly. For passaging, organoids were collected using organoid washing solution (Daxiang Technology, KC100141), digested with organoid digestion solution (Daxiang Technology, KC100145), centrifuged, and re‐embedded in matrix gel for continued culture.

For lentiviral infection, organoids were gently dissociated into small clusters and incubated with lentivirus carrying either an empty vector or an engineered partial BRCA1 construct containing the 2594delC‐associated mutant sequence and an N‐terminal 4 × FLAG tag in organoid culture medium. After infection, organoids were re‐embedded in matrix gel and cultured in fresh organoid medium. The expression of the FLAG‐tagged engineered partial BRCA1 construct was verified by Western blotting, and successfully infected organoids were used for subsequent uptake, viability, immunofluorescence, ELISA, and ATP assays.

### 4.3 Synthesis of Folate‐PEG_2_K‐ssDNA

Folate‐PEG_2_K‐ssDNA was synthesized through an amide coupling reaction between NHS‐activated Folate‐PEG_2_K and amino‐modified ssDNA. Briefly, Folate‐PEG_2_K‐NHS and 5’‐amino‐modified ssDNA complementary to the anchoring sequence of S1 were dissolved in DMSO/H_2_O in the presence of TEA. The reaction mixture was stirred at room temperature in the dark to allow conjugation between the NHS ester and the terminal amino group of ssDNA. The resulting Folate‐PEG_2_K‐ssDNA was purified by C18 reverse‐phase HPLC and verified by MALDI‐TOF MS. After lyophilization, the purified Folate‐PEG_2_K‐ssDNA was stored at 4°C in the dark for subsequent tFNA assembly.

### 4.4 Synthesis of Olaparib‐SS‐ssDNA

Olaparib‐SS‐ssDNA was prepared through a reduction‐responsive disulfide linker strategy. Olaparib was first reacted with N‐Boc‐3‐bromopropylamine under alkaline conditions to yield Compound 1, followed by Boc deprotection with trifluoroacetic acid (TFA)/dichloromethane (DCM) to afford the primary amine intermediate Compound 2. Compound 2 was coupled with a disulfide‐containing dicarboxyl linker (COOH‐SS‐COOH) by EDC/NHS‐mediated amide condensation to obtain Olaparib‐SS‐COOH. Subsequently, Olaparib‐SS‐COOH was reacted with N‐(2‐aminoethyl)maleimide in the presence of TEA in DMSO/H_2_O to introduce the maleimide group, yielding Olaparib‐SS‐MAL. Finally, Olaparib‐SS‐MAL was conjugated to the 5’‐thiol‐modified S3 DNA strand (5’‐SH‐ssDNA) via maleimide‐thiol coupling, and the product was purified by HPLC to obtain Olaparib‐SS‐ssDNA for subsequent tFNA assembly.

### 4.5 Synthesis of Adavosertib‐SS‐ssDNA

Adavosertib‐SS‐ssDNA was synthesized through a reduction‐responsive disulfide linker and maleimide‐thiol conjugation strategy. Briefly, adavosertib was first reacted with N‐Boc‐3‐bromopropylamine under alkaline conditions to yield Compound 1, followed by Boc deprotection with trifluoroacetic acid/dichloromethane (TFA/DCM) to afford the primary amine intermediate Compound 2. Compound 2 was then coupled with a disulfide‐containing dicarboxyl linker (COOH‐SS‐COOH) via EDC/NHS‐mediated amide condensation to obtain Adavosertib‐SS‐COOH. Subsequently, Adavosertib‐SS‐COOH was reacted with N‐(2‐aminoethyl)maleimide to introduce a terminal maleimide group, yielding Adavosertib‐SS‐MAL. Finally, Adavosertib‐SS‐MAL was conjugated to the 5’‐thiol‐modified S4 DNA strand via maleimide‐thiol coupling. The product was purified by HPLC to obtain Adavosertib‐SS‐ssDNA for subsequent tFNA assembly. Key intermediates and final products were characterized by ^1^H NMR and MALDI‐TOF MS.

### 4.6 Design and Synthesis of Tetrahedral Framework Nucleic Acids

The tFNA scaffold was self‐assembled from four 87‐nt single‐stranded DNA strands (S1‐S4), each comprising a 72‐nt framework region and a 15‐nt anchoring region. For FBOA‐tFNA construction, S1 was modified with a folate‐mediated targeting module; S2 was engineered with a BRCA1 mutation‐specific molecular beacon; S3 was conjugated with a disulfide‐linked olaparib module (Olaparib‐SS); and S4 was conjugated with a disulfide‐linked WEE1 inhibitor module (Adavosertib‐SS). Human BRCA1 2594delC‐ and murine BRCA1 972delG‐responsive beacon variants were used for the UWB1.289/organoid and ID8‐BRCA1‐mut models, respectively. In fluorescently labeled variants, Cy5 was incorporated to track folate‐mediated uptake, and the molecular beacon was labeled with FAM/BHQ1 to monitor mutation‐responsive activation. Fluorescently labeled FBO‐tFNA or FBOA‐tFNA was used only for cellular uptake, mutation‐responsive activation, and in vivo targeting/biodistribution studies. For therapeutic efficacy, cytotoxicity, Western blotting, ELISA, flow cytometry‐based immune analysis, and repeated‐dose tolerability experiments, nonfluorescent FBO‐tFNA, FBA‐tFNA, or FBOA‐tFNA counterparts with the same framework, targeting ligand, mutation‐responsive sequence, and corresponding drug‐conjugated modules but without Cy5/FAM labeling were used unless otherwise indicated. FBO‐tFNA and FBA‐tFNA were prepared as single‐drug control nanoplatforms by omitting the adavosertib or olaparib module, respectively. All DNA strands were synthesized by Tsingke Biotech, HPLC‐purified, and verified by MALDI‐TOF MS.

For tFNA assembly, S1‐S4 were mixed at equimolar ratios to a final concentration of 1 µM in TAE‐Mg^2^^+^ buffer containing 12.5 mM MgCl_2_. The mixture was denatured at 95°C for 10 min and gradually cooled to 4°C at 1°C per 30 s to form tetrahedral structures. Assembly efficiency was assessed by 15% native polyacrylamide gel electrophoresis (native PAGE). Functional modules were introduced by stepwise hybridization. Depending on the experimental design, preassembled tFNA was sequentially incubated with the corresponding folate or Folate‐Cy5 targeting module, an unlabeled or FAM/BHQ1‐labeled BRCA1 molecular beacon, Olaparib‐SS‐Duplex, and/or Adavosertib‐SS‐Duplex at molar ratios of 1:1.2‐1.5 at 25°C for 2–4 h. After each hybridization step, products were purified using 100 kDa ultrafiltration tubes to remove unbound components. The resulting FBO‐tFNA, FBA‐tFNA, and FBOA‐tFNA formulations were stored at 4°C in the dark and used within one week.

### 4.7 Native Polyacrylamide Gel Electrophoresis (PAGE) Analysis

Stepwise tFNA assembly was verified by 15% native polyacrylamide gel electrophoresis (PAGE; 29:1 acrylamide/bis‐acrylamide). Each assembly intermediate, including partially hybridized products and intact tFNA, was mixed with 6× loading buffer (Thermo Fisher, R0611) before loading. Electrophoresis was performed in 1 × TAE‐Mg^2^^+^ buffer (40 mM Tris‐acetate, 1 mM EDTA, 12.5 mM MgCl_2_, pH 8.0) at 80 V for 90 min. The gel was stained with GelRed nucleic acid dye (Shanghai Life‐iLab Biotech Co., Ltd, AN34L022) in the dark for 30 min and imaged using a gel imaging system. Assembly efficiency and functional module integration were assessed by comparing the migration shifts and apparent size increases of different intermediates.

### 4.8 Nuclease and Serum Stability Evaluation

To evaluate nuclease resistance and serum stability, tFNA (100 nmol L^−1)^ was incubated with DNase I (10 U mL^−1^; AmBeed, A2949013) at 37°C or maintained in complete medium containing 10% fetal bovine serum (FBS) at 37°C. Samples were collected at 0, 2, 4, 6, and 12 h. For DNase I‐treated samples, ethylenediaminetetraacetic acid (EDTA) was immediately added to a final concentration of 20 mmol L^−1^ to terminate digestion. For serum‐treated samples, serum nucleases were inactivated by heating at 95°C for 5 min. Samples from each time point were separated by 15% native polyacrylamide gel electrophoresis (PAGE), stained with GelRed, and imaged to assess the structural integrity of tFNA under biological conditions.

### 4.9 Transmission Electron Microscopy (TEM) Characterization

tFNA was diluted to 50 nM, dropped onto carbon‐coated copper grids (Zhongjing Keyi, BZ11023a), and adsorbed at room temperature for 5 min. The grids were negatively stained with 2% phosphotungstic acid (pH 7.0) for 90 s and air‐dried naturally. tFNA morphology was observed using a Hitachi HT7700 transmission electron microscope at 100 kV, and representative images were collected. Diameters of at least 100 randomly selected nanoparticles were measured with ImageJ to calculate the mean particle size and size distribution.

### 4.10 Dynamic Light Scattering (DLS) and Zeta Potential Measurement

tFNA was diluted to 500 nM and filtered through a 0.22 µm membrane before analysis using a Zetasizer Nano particle analyzer (Malvern Instruments Ltd., England). DLS was performed at 25°C with a 90° scattering angle and 120 s equilibration time. Each sample was measured three times, with 10–15 sub‐runs per measurement, to determine the hydrodynamic diameter (Dh) and polydispersity index (PDI). Zeta potential was measured at 25°C under an electric field strength of 20 V cm^−1^ and calculated using the Smoluchowski model.

### 4.11 Dual‐Drug Loading Efficiency and Site‐Defined Drug Incorporation Analysis

The loading efficiencies of olaparib and adavosertib in tFNA were determined by high‐performance liquid chromatography (HPLC). Briefly, the assembled tFNA suspension was transferred to a 10 kDa centrifugal ultrafiltration tube and centrifuged at 12,000 × g for 20 min at 4°C to separate tFNA‐associated drug from unbound drug. The filtrate was collected, together with all washing fractions when additional washing steps were performed. The amount of free olaparib or adavosertib in the filtrate and washing fractions was quantified by HPLC.

HPLC analysis was performed using a Waters 2695 system equipped with a C18 reversed‐phase column. The mobile phase consisted of acetonitrile and 0.1% aqueous formic acid (35:65, v/v) at a flow rate of 0.8 mL min^−^^1^. Olaparib and adavosertib were detected at 234 nm, and the column temperature was maintained at 30°C. Independent calibration curves were established for olaparib and adavosertib over the tested concentration range. The corresponding linear regression equations were y = 68517x + 71607 (R^2^ = 0.9998) for olaparib and y = 60385x + 31830 (R^2^ = 0.9999) for adavosertib, with retention times of approximately 6.319 and 6.768 min, respectively. Three independent batches were analyzed for loading efficiency.

The amount of loaded drug was calculated by subtracting the amount of free drug in the filtrate and washing fractions from the initial amount added. The loading efficiency (LE) was calculated as follows: LE (%) = (M_input − M_unbound) / M_input × 100%, where M_input represents the initial mass of drug added, and M_unbound represents the total mass of free drug detected in the filtrate and washing fractions. To determine the site‐defined incorporation capacity, drug‐containing modules were introduced at 1.0, 1.5, and 2.0 equivalents per tFNA, and the number of incorporated olaparib and adavosertib molecules per tFNA was calculated from the HPLC‐determined drug amount and tFNA concentration.

### 4.12 LC–MS/MS Analysis of Free and Linker‐Bound Drug Species

LC–MS/MS was performed to distinguish free olaparib/adavosertib from linker‐associated drug forms. FBOA‐tFNA samples were incubated in PBS or 10 mM GSH‐containing buffer at 37°C. After incubation, samples were analyzed by LC–MS/MS following chromatographic separation and mass spectrometric detection. Free drug and linker‐bound drug species were identified according to their retention times and mass‐to‐charge ratios. The relative fractions of free and linker‐associated drugs were calculated based on integrated peak areas of the corresponding LC–MS/MS signals.

### 4.13 Reduction‐Responsive Drug Release and Non‐Cleavable Linker Control

Reduction‐responsive drug release was evaluated by incubating the cleavable olaparib‐ and adavosertib‐containing constructs in PBS with or without 10 mM GSH at 37°C. Samples were collected at 0, 2, 8, and 24 h, and the released olaparib and adavosertib were quantified by HPLC using the corresponding calibration curves. Cumulative drug release was calculated relative to the total incorporated drug amount.

To verify that drug liberation depended on disulfide‐bond cleavage, corresponding non‐cleavable control constructs were prepared by replacing the reduction‐responsive disulfide linker with a non‐cleavable linker while retaining the remaining drug‐containing module. These constructs were analyzed under the same PBS and 10 mM GSH conditions. The cleavable constructs showed markedly enhanced drug release in the presence of GSH, whereas release from the non‐cleavable controls remained low, supporting a linker‐dependent reduction‐responsive drug‐liberation mechanism.

### 4.14 Construction of the Engineered Partial BRCA1^2594delC^‐Containing Expression Model

Patient‐derived ovarian cancer organoids were transduced with an engineered partial BRCA1 construct containing the 2594delC‐associated mutant sequence; empty‐vector‐transduced organoids served as controls. The construct comprised a 1,047‐bp coding sequence with an N‐terminal 4×FLAG tag, GS linker, and partial BRCA1‐derived sequence, encoding a predicted 348‐amino‐acid product of ∼39.1 kDa. The complete 1,047‐bp coding sequence was verified by Sanger sequencing and matched the designed sequence, including the intended mutation region. This mutant‐transcript system was used to evaluate BRCA1 mutation‐responsive molecular recognition rather than endogenous BRCA1 loss‐of‐function.

### 4.15 Cellular Uptake Assay

For fluorescence‐based uptake and activation assays, fluorescently labeled FBO‐tFNA or FBOA‐tFNA was used, with Cy5 tracking folate‐mediated uptake and FAM reporting molecular beacon activation. UWB1.289 cells were seeded in confocal dishes at 1 × 10^5^ cells/dish or in 6‐well plates at 5 × 10^5^ cells/well and cultured at 37°C for 24 h. Ovarian cancer organoids transduced with an empty vector or the engineered partial BRCA1^2594delC^‐containing construct were seeded in confocal‐compatible dishes. Cells or organoids were incubated with fluorescently labeled FBO‐tFNA or FBOA‐tFNA (100 nM), and Cy5 and FAM fluorescence were monitored by confocal microscopy at predefined time intervals to evaluate folate‐mediated uptake and BRCA1 mutant‐transcript‐responsive molecular‐beacon activation. Nuclei were stained with Hoechst 33342 (Beijing Solarbio Science & Technology Co., Ltd., C0031; 5 µg mL^−^^1^) when required. For organoids, Cy5 and FAM fluorescence intensities were quantified from confocal images using ImageJ.

For flow cytometry, cells were washed with cold PBS, while organoids were gently dissociated into single‐cell suspensions. Samples were resuspended in PBS containing 2% FBS and analyzed using a Beckman CytoFLEX Flow Cytometer. Surface FOLR1 expression in HeLa, SK‐OV‐3, UWB1.289, and UWB1.289‐BRCA1‐restored cells was evaluated by flow cytometry using an anti‐FOLR1 antibody. To verify folate‐targeting ability and BRCA1 mutation‐recognition specificity, UWB1.289, UWB1.289‐BRCA1‐restored, SK‐OV‐3, and HeLa cells were comparatively analyzed after incubation with FBO‐tFNA. For the folate competition assay, UWB1.289 cells were preincubated with excess free folic acid before FBOA‐tFNA treatment, followed by flow‐cytometric analysis of Cy5 fluorescence. Fluorescence‐positive rates, Cy5/FAM mean fluorescence intensities, and colocalization were used to evaluate targeted uptake and mutation‐responsive molecular recognition.

### 4.16 Construction and Validation of the Scrambled Molecular‐Beacon Control

To evaluate the sequence specificity of BRCA1 mutant‐transcript recognition, a scrambled molecular‐beacon control (F‐scrMB‐O‐tFNA) was constructed by replacing the BRCA1‐responsive beacon sequence with a nonresponsive scrambled sequence while retaining the folate‐targeting and olaparib‐containing modules. UWB1.289 and UWB1.289‐BRCA1‐restored cells were incubated with fluorescently labeled F‐scrMB‐O‐tFNA or FBO‐tFNA under identical conditions. Cy5 fluorescence was used to evaluate cellular uptake, whereas FAM fluorescence was used to assess molecular‐beacon activation. Confocal images were acquired, and fluorescence intensities were quantified using ImageJ. For functional comparison, cell viability after treatment with PBS, blank tFNA, drug‐free FB‐tFNA, F‐scrMB‐O‐tFNA, or FBO‐tFNA was determined by CCK‐8 assay as described below.

### 4.17 Intracellular Olaparib‐Associated Exposure Kinetics

UWB1.289 cells were treated with free olaparib or FBO‐tFNA at matched olaparib‐equivalent concentrations. Cells were collected at the indicated time points from 1 to 48 h, washed thoroughly with cold PBS to remove extracellular drug, and lysed for HPLC analysis. Intracellular olaparib‐associated levels were quantified using the established olaparib calibration curve and normalized to the corresponding cell samples.

The temporal intracellular profiles of free olaparib and FBO‐tFNA were then compared. Because the HPLC assay quantifies total intracellular olaparib‐associated content without chemically distinguishing released free olaparib from residual linker‐associated drug, these measurements were interpreted as intracellular olaparib‐associated exposure rather than direct intracellular release kinetics.

### 4.18 Organoid Live/Dead Staining Assay

Organoid viability after different treatments was evaluated using a Calcein‐AM/PI Double Staining Kit (AbMole, M55257). Briefly, ovarian cancer organoids were seeded in confocal‐compatible dishes and treated with the indicated formulations. After treatment, organoids were gently washed with PBS and incubated with freshly prepared Calcein‐AM/PI working solution at 37°C in the dark for 30 min. After staining, live and dead cells were visualized by confocal microscopy, with Calcein‐AM‐positive live cells shown as green fluorescence and PI‐positive dead cells shown as red fluorescence. Fluorescence images were acquired using a confocal laser scanning microscope.

### 4.19 Organoid ATP Viability Assay

Organoid viability was assessed using an organoid ATP activity assay kit (Orgen Biotech, CP‐00810) according to the manufacturer's instructions. Briefly, ovarian cancer organoids were seeded in 96‐well plates and treated with the indicated formulations. After treatment, plates were equilibrated to room temperature for 30 min, and an equal volume of ATP detection reagent was added to each well. The plates were vigorously shaken for 5 min to fully lyse the organoids, followed by incubation at room temperature for 25 min to stabilize the luminescent signal. Luminescence was measured using a multifunctional microplate reader, and organoid viability was calculated relative to the control group.

### 4.20 Cell Viability Assay

The cytotoxicity of FBO‐tFNA and FBOA‐tFNA against BRCA1‐mutant and BRCA1‐wild‐type ovarian cancer cells was evaluated using the CCK‐8 assay. UWB1.289, UWB1.289‐BRCA1‐restored, and ID8‐BRCA1‐mut cells were seeded in 96‐well plates at 5 × 10^3^ cells/well and cultured for 24 h. Cells were then treated with blank tFNA, free olaparib, free olaparib plus adavosertib (Ola+Ada), FBO‐tFNA, or FBOA‐tFNA for 48 h. Drug concentrations were calculated as olaparib equivalents and ranged from 0.1 to 100 µM. After treatment, 10 µL CCK‐8 solution (Shanghai Life‐iLab Biotech Co., Ltd, AC11L054) was added to each well, followed by incubation at 37°C for 2 h. Absorbance was measured at 450 nm using a microplate reader. Cell viability was normalized to the PBS group. Concentration‐response curves were fitted by nonlinear regression using GraphPad Prism, and IC_50_ values were calculated. For time‐dependent assays, cells were treated with 10 µM drug equivalent for different durations, and viability was determined as above.

### 4.21 Drug Combination Synergy and Intracellular Drug Quantification

To quantify the pharmacological interaction between PARP and WEE1 inhibition, UWB1.289 cells were treated with dose‐response matrices of free olaparib plus adavosertib or FBO‐tFNA plus adavosertib for 48 h. Cell viability was measured by the CCK‐8 assay, and drug interactions were evaluated using the Bliss independence model. Positive Bliss scores were interpreted as synergistic interactions.

For intracellular drug quantification, UWB1.289 cells were treated with free Ola+Ada, FBO‐tFNA + FBA‐tFNA, or FBOA‐tFNA at matched extracellular drug doses. Cells were collected at 1, 4, and 8 h, washed thoroughly with PBS, and lysed for HPLC analysis. Intracellular olaparib‐ and adavosertib‐associated levels were quantified using their respective calibration curves, and the intracellular olaparib‐to‐adavosertib (Ola/Ada) molar ratio was calculated at each time point to evaluate temporal coordination of dual‐drug exposure.

### 4.22 Intracellular GSH/GSSG Measurement and BSO Pretreatment Assay

UWB1.289 cells were seeded in 6‐well plates at 5 × 10^5^ cells per well and cultured for 24 h before treatment with PBS, FBO‐tFNA, free olaparib plus adavosertib (Ola+Ada), or FBOA‐tFNA at an olaparib‐equivalent concentration of 10 µM for 6 h. Intracellular glutathione levels were measured using a GSH and GSSG Assay Kit (TO1050L; Leagene Biotechnology, Beijing, China) according to the manufacturer's instructions. Cells were collected, rapidly lysed, and deproteinized under low‐temperature conditions to minimize artificial oxidation of GSH. Total glutathione and GSSG contents were determined using a microplate reader, and reduced GSH was calculated as total glutathione minus two times the GSSG content. Reduced GSH and GSSG contents were normalized to the total protein content of the corresponding samples, and the intracellular GSH/GSSG ratio was subsequently calculated.

For GSH‐depletion experiments, UWB1.289 cells were pretreated with buthionine sulfoximine (BSO; AmBeed, A215764, 100 µM) for 24 h and subsequently treated with FBOA‐tFNA at an olaparib‐equivalent concentration of 10 µM for 48 h in the continued presence of BSO. The experimental groups included PBS, BSO, FBOA‐tFNA, and BSO plus FBOA‐tFNA. Cell viability was determined using the CCK‐8 assay as described above and normalized to the PBS‐treated group. The selected BSO condition reduced intracellular glutathione while exerting minimal cytotoxicity when administered alone.

### 4.23 Western Blot Analysis

UWB1.289 or ID8‐BRCA1‐mut cells were seeded in 6‐well plates at 1 × 10^6^ cells/well and cultured at 37°C for 12 h. Cells were treated with PBS, olaparib, FBO‐tFNA, Ola+Ada, or FBOA‐tFNA at 20 µM olaparib equivalent for 6 h, washed three times with cold PBS, and cultured in complete medium containing 10% FBS for another 18 h. To evaluate concentration‐ and time‐dependent changes in DNA damage and homologous recombination repair markers, cells were treated with the indicated formulations across predefined concentration gradients or predefined time‐course gradients, followed by Western blot analysis. For organoid experiments, ovarian cancer organoids were treated with the indicated formulations, collected, and lysed using the same procedure. Samples were lysed with RIPA buffer containing protease and phosphatase inhibitors (Shanghai Life‐iLab Biotech Co., Ltd, AP02L192), followed by centrifugation at 12 000 rpm for 15 min. Protein concentrations were measured using a BCA assay kit (Shanghai Life‐iLab Biotech Co., Ltd, AP12L025). Equal amounts of protein, 30 µg per sample, were separated by SDS‐PAGE and transferred onto PVDF membranes (Shanghai Life‐iLab Biotech Co., Ltd, AG14L022/023). After blocking, membranes were incubated overnight at 4°C with primary antibodies against BRCA1 (Proteintech, 83390‐6‐RR, RRID: AB_3671044), FOLR1 (HUABIO, HA722768), 53BP1 (Proteintech, 20002‐1‐AP, RRID: AB_3085592), RAD51 (Proteintech, 67024‐1‐Ig, RRID: AB_2882339), γ‐H2AX (HUABIO, ET1602‐2), WEE1 (Proteintech, 29474‐1‐AP, RRID: AB_2918314), p‐Chk1 Ser345 (HUABIO, HA721189), p‐CDK1 Tyr15 (Proteintech, 82751‐5‐RR, RRID: AB_3743425), STING (HUABIO, HA722832), p‐STING Ser366 (HUABIO, HA723137), p‐STING Ser365 (CST, #72971), TBK1 (HUABIO, HA723365), p‐TBK1 Ser172 (Abways, CY8364), IRF3 (HUABIO, ET1612‐14), p‐IRF3 Ser396 (HUABIO, HA722772), active + pro caspase‐3 (Nature Biosciences, A71383), and β‐actin (HUABIO, HA722023). Membranes were washed five times and incubated with HRP‐conjugated secondary antibodies at room temperature for 1 h. Bands were visualized with ECL reagent (Beyotime, P0018AS) and imaged using an Amersham Imager 600 system. STING, TBK1, and IRF3 phosphorylation were quantified as the corresponding phospho/total protein ratios, while caspase‐3 activation was quantified as the cleaved caspase‐3/total caspase‐3 ratio. β‐actin was used as the loading control.

### 4.24 Evaluation of Apoptosis, Colony Formation, and Migration after tFNA Treatment

Apoptosis after FBO‐tFNA and FBOA‐tFNA treatment was assessed by Annexin V‐FITC/PI double‐staining flow cytometry. UWB1.289, UWB1.289‐BRCA1‐restored, and ID8‐BRCA1‐mut cells were seeded in 6‐well plates at 2 × 10^5^ cells/well and cultured at 37°C for 24 h. Cells were treated with PBS, free olaparib (20 µM), free olaparib plus adavosertib (20 µM each), tFNA, FBO‐tFNA, or FBOA‐tFNA, with tFNA formulations normalized to 20 µM olaparib equivalent, for 48 h. Floating cells and adherent cells detached with 0.25% trypsin without EDTA were collected, combined, and washed twice with cold PBS. A total of 1 × 10^5^ cells were resuspended in 100 µL 1× binding buffer and stained with 5 µL Annexin V‐FITC and 5 µL PI (Shanghai Life‐iLab Biotech Co., Ltd, AC12L033) at room temperature in the dark for 15 min. Then, 400 µL binding buffer was added, and samples were analyzed within 1 h using a Beckman CytoFLEX Flow Cytometer. Data were analyzed with FlowJo, and early apoptotic cells (Annexin V^+^/PI^−^) and late apoptotic cells (Annexin V^+^/PI^+^) were quantified.

For colony formation assays, UWB1.289 and ID8‐BRCA1‐mut cells were treated with the indicated formulations for 48 h, trypsinized, counted, and reseeded in 6‐well plates at 500 cells/well with 2 mL complete medium containing 10% FBS. Cells were cultured at 37°C with 5% CO_2_ for 10–14 days, with medium replaced every 3 days. After visible colonies formed, cells were washed twice with cold PBS, fixed with methanol for 15 min, and stained with 0.1% crystal violet (Shanghai Life‐iLab Biotech Co., Ltd, AS11L112) at room temperature for 30 min. Excess dye was removed under running water. After air‐drying, images were captured, and colony numbers were quantified using ImageJ.

A wound‐healing assay was used to assess cell migration. ID8‐BRCA1‐mut cells were seeded in 6‐well plates at 5 × 10^5^ cells/well and cultured to 90%‐95% confluence. A linear scratch was generated across the monolayer using a sterile 200 µL pipette tip, and detached cells were removed by washing three times with cold PBS. To minimize the potential contribution of cell proliferation to wound closure, cells were subsequently cultured in serum‐free or low‐serum medium containing 0.5%‐1% FBS. Different drug formulations were added at the concentrations described above, and cells were cultured at 37°C with 5% CO_2_ under the same serum‐restricted conditions. Bright‐field images were acquired at 0 and 48 h using a Leica S9i stereofluorescence microscope at 40× magnification. Migration efficiency was calculated using ImageJ.

### 4.25 ELISA Analysis

Organoid culture supernatants, cell culture supernatants, or tumor tissue homogenates were collected after the indicated treatments and analyzed using commercial ELISA kits according to the manufacturers’ instructions. Briefly, culture supernatants were centrifuged at 1000 × g for 20 min to remove cell debris, and the clarified supernatants were collected for ELISA analysis. Tumor tissues were weighed, homogenized in RIPA lysis buffer (Shanghai Life‐iLab Biotech Co., Ltd, AP01L013) containing protease inhibitors, and centrifuged at 12 000 × g for 15 min at 4°C; the supernatants were collected and normalized according to protein concentration. ELISA kits were used to detect HMGB1 (Aimeng Youning, Shanghai, China, LV10792M), human IFN‐β (Jianglai Bio, JL19215), human IL‐6 (Jianglai Bio, JL14113), poly(ADP‐ribose) (Wuhan Fine Biotech, EU2597), mouse IFN‐β (Aimeng Youning, Shanghai, China, LV30252M), mouse IL‐6 (Aimeng Youning, Shanghai, China, LV30325M), and TNF‐α (Aimeng Youning, Shanghai, China, LV30536M). Absorbance was measured at 450 nm using a microplate reader, and analyte concentrations were calculated from standard curves.

### 4.26 Cell Cycle Analysis

Flow cytometry was used to assess cell‐cycle distribution after treatment. ID8‐BRCA1‐mut cells were seeded in 6‐well plates at 2 × 10^5^ cells/well. After 24 h, cells were treated with PBS, FBO‐tFNA, free olaparib plus adavosertib, or FBOA‐tFNA, with drug concentrations normalized to 20 µM olaparib equivalent. After 48 h, cells were collected, washed twice with cold PBS, and fixed in prechilled 70% ethanol at 4°C overnight. Fixed cells were washed with PBS and stained using a Cell Cycle Assay Kit (Elabscience, E‐CK‐A351) at room temperature in the dark for 30 min. DNA content was detected using a Beckman CytoFLEX Flow Cytometer, and the proportions of cells in G1, S, and G2/M phases were analyzed with FlowJo.

### 4.27 Determination of γ‐H2AX and Intracellular dsDNA Signals

Immunofluorescence staining was performed to detect intracellular γ‐H2AX foci and dsDNA signals. UWB1.289 or ID8‐BRCA1‐mut cells were seeded in confocal dishes at 1 × 10^5^ cells/dish. After 24 h, cells were treated with the indicated formulations. At the end of treatment, cells were washed with cold PBS, fixed with 4% paraformaldehyde (Shanghai Life‐iLab Biotech Co., Ltd, AC28L112) for 15 min, permeabilized with 0.3% Triton X‐100 for 10 min, and blocked with 5% BSA (Shanghai Life‐iLab Biotech Co., Ltd, AP36L015) for 1 h. For γ‐H2AX staining, cells were incubated with anti‐γ‐H2AX antibody (HUABIO, ET1602‐2, 1:400) overnight at 4°C, followed by incubation with Alexa Fluor 488‐ or Alexa Fluor 555‐conjugated secondary antibodies (1:500) at room temperature in the dark for 1 h. For intracellular dsDNA signal detection, cells were incubated with Quant‐iT PicoGreen dsDNA Assay Kit (Thermo Scientific, P7589) according to the manufacturer's instructions. Nuclei were counterstained with DAPI. Images were acquired using a confocal laser scanning microscope (TCS SP8/DMIL LED/S9I/DM2500; Leica, Germany). γ‐H2AX foci per nucleus and intracellular dsDNA fluorescence intensity were quantified using ImageJ.

### 4.28 Detection of p‐STING, HMGB1, and PD‐L1 Expression

Immunofluorescence staining was performed to detect p‐STING, HMGB1, and PD‐L1 expression and localization. ID8‐BRCA1‐mut cells were seeded in confocal dishes at 1 × 10^5^ cells/dish. After treatment, cells were washed with cold PBS, fixed with 4% paraformaldehyde for 15 min, permeabilized with 0.3% Triton X‐100 for 10 min, and blocked with 5% BSA for 1 h. Cells were incubated overnight at 4°C with anti‐p‐STING antibody (HUABIO, HA723137, 1:200), anti‐HMGB1 antibody (Nature Biosciences, A90225, 1:200), or anti‐PD‐L1 antibody (Nature Biosciences, A78758, 1:200). After PBS washing, cells were incubated with Alexa Fluor 488‐ or Alexa Fluor 555‐conjugated secondary antibodies (1:500) at room temperature in the dark for 1 h, followed by DAPI staining (Shanghai Life‐iLab Biotech Co., Ltd, AS21L122). Images were acquired using a confocal laser scanning microscope (TCS SP8/DMIL LED/S9I/DM2500; Leica, Germany), and fluorescence intensities were quantified with ImageJ. For HMGB1 subcellular distribution analysis, nuclear and cytoplasmic regions were defined using DAPI staining, and the cytoplasmic‐to‐nuclear HMGB1 fluorescence ratio was calculated. Identical analysis settings were applied across all groups.

### 4.29 Quantitative Real‐Time PCR

qPCR was performed to determine immune‐related gene expression after treatment. ID8‐BRCA1‐mut cells were seeded in 6‐well plates at 5 × 10^5^ cells/well. After 24 h, cells were treated with the indicated formulations for 48 h. Cells were washed twice with cold PBS, and total RNA was extracted using TRIzol reagent (ABKBio, ABK0005M). RNA concentration and purity were measured using a NanoDrop 2000 spectrophotometer. Total RNA, 1 µg, was reverse‐transcribed into cDNA using PrimeScript RT Master Mix (Takara, RR036A). qPCR was performed using PerfectStart Green qPCR SuperMix (TransGen Biotech, AQ602) on a QuantStudio 5 Real‐Time PCR System (Thermo Fisher, Singapore). Relative gene expression was calculated using the 2^−^ΔΔCt method, with 18S as the internal reference. Target genes included CXCL10, CCL5, IFN‐β, and PD‐L1. Primers were synthesized by Tsingke Biotech, and sequences were as follows:

CXCL10 forward: 5’‐AATGAGGGCCATAGGGAAGC‐3’;
CXCL10 reverse: 5’‐GGATTCAGACATCTCTGCTCATC‐3’;
CCL5 forward: 5’‐ATATGGCTCGGACACCACTC‐3’;
CCL5 reverse: 5’‐CAAACACGACTGCAAGATTGG‐3’;
IFN‐β forward: 5’‐TTACACTGCCTTTGCCATCC‐3’;
IFN‐β reverse: 5’‐AGAAACACTGTCTGCTGGTG‐3’;
PD‐L1 forward: 5’‐ATGCCCCATACCGCAAAATC‐3’;
PD‐L1 reverse: 5’‐TGACACTTCTCTTCCCACTCAC‐3’;
18S forward: 5’‐AGAAACGGCTACCACATCCA‐3’;
18S reverse: 5’‐CACCAGACTTGCCCTCCA‐3’.

### 4.30 In Vitro Activation of Dendritic Cells

Bone marrow cells were isolated from the femurs and tibias of 6‐week‐old female C57BL/6 mice. After ACK red blood cell lysis, cells were resuspended in high‐glucose DMEM supplemented with 10% FBS, 20 ng mL^−^^1^ GM‐CSF (Thermo Fisher, 315‐03), and 10 ng mL^−^^1^ IL‐4 (Thermo Fisher, 214‐14), and cultured at 37°C in 5% CO_2_ for 6 days to generate immature bone marrow‐derived dendritic cells (BMDCs). Half of the culture medium was replaced every 2 days.

ID8‐BRCA1‐mut cells were seeded in 6‐well plates at 1 × 10^5^ cells per well and treated with PBS, FBO‐tFNA, free olaparib plus adavosertib (Ola+Ada), or FBOA‐tFNA, with drug concentrations normalized to 20 µM olaparib equivalent. After 48 h of treatment, drug‐treated ID8‐BRCA1‐mut cells were collected, washed with PBS, and transferred to the upper chambers of 0.4 µm Transwell inserts. Immature BMDCs were seeded in the corresponding lower chambers at 1 × 10^6^ cells per well. Tumor cells and BMDCs were co‐cultured for an additional 24 h to allow paracrine communication without direct cell‐cell contact. BMDCs were then collected from the lower chambers and stained with anti‐CD11c‐APC (Elabscience, E‐AB‐F0991E), anti‐CD80‐PE (Elabscience, E‐AB‐F0992D, RRID: AB_3713131), and anti‐CD86‐FITC (Bioss, bsm‐30162A‐FITC) at 4°C in the dark for 30 min. Flow cytometry was performed to determine the proportion of CD80^+^CD86^+^ mature dendritic cells within the CD11c^+^ population.

### 4.31 Pharmacological STING Inhibition and STING‐Dependent BMDC Maturation Assay

To evaluate the contribution of STING signaling to FBOA‐tFNA‐induced immune activation, ID8‐BRCA1‐mut cells were assigned to four groups: PBS, STING inhibitor, FBOA‐tFNA, and FBOA‐tFNA plus STING inhibitor. Cells were treated with C‐176 (AbMole, M9893, 1 µM) for 30 min and subsequently exposed to FBOA‐tFNA under the indicated conditions. Protein lysates were collected for Western blot analysis of total and phosphorylated STING, TBK1, and IRF3. Phosphorylation levels were quantified as p‐STING/STING, p‐TBK1/TBK1, and p‐IRF3/IRF3 ratios.

For functional validation, treated tumor cells were subsequently used in the tumor cell–BMDC Transwell coculture assay. After coculture, BMDCs were collected and stained for CD11c, CD80, and CD86, and the proportion of CD11c^+^CD80^+^CD86^+^ mature BMDCs was quantified by flow cytometry. The effect of pharmacological STING inhibition on FBOA‐tFNA‐induced BMDC maturation was used to assess the STING‐dependent component of the immunostimulatory response.

### 4.32 T‐Cell Activation Analysis

A Transwell co‐culture system was used to evaluate T‐cell activation induced by drug‐treated tumor cells. CD3^+^ T cells were isolated from spleens of 6‐week‐old female C57BL/6 mice by positive selection using mouse CD3 MicroBeads (Miltenyi Biotec, 130‐094‐973). ID8‐BRCA1‐mut cells were seeded in the lower chambers of Transwell inserts (Corning, 3412; 0.4 µm pore size) at 2 × 10^5^ cells/well and treated with PBS, free olaparib plus adavosertib, FBO‐tFNA, or FBOA‐tFNA, with drug concentrations normalized to 20 µM olaparib equivalent. After 48 h, isolated CD3^+^ T cells were added to the upper chambers at 1 × 10^6^ cells/well and co‐cultured for 24 h. T cells were collected and stained with anti‐CD3‐FITC (Elabscience, E‐AB‐F1013C, RRID: AB_3065041), anti‐CD25‐APC (Elabscience, E‐AB‐F1102E, RRID: AB_3661685), and anti‐CD69‐PE (Elabscience, E‐AB‐F1187D, RRID: AB_3712293) at 4°C in the dark for 30 min. The proportions of CD25^+^ and CD69^+^ cells among CD3^+^ T cells were determined by flow cytometry.

### 4.33 RNA‐seq Analysis of FBOA‐tFNA‐Treated Cells

To assess transcriptomic changes induced by FBOA‐tFNA in BRCA1‐mutant ovarian cancer cells, ID8‐BRCA1‐mut cells were seeded in 6‐well plates at 1 × 10^6^ cells/well. After 12 h, cells were treated with PBS or FBOA‐tFNA, with the concentration normalized to 20 µM olaparib equivalent. After 24 h, cells were collected, and total RNA was extracted using TRIzol. Three biological replicates were included per group. After RNA concentration, purity, and integrity assessment, qualified RNA samples were sent to Wuhan iGeneBook Biotechnology Co., Ltd. for library construction and RNA sequencing. Raw data were quality‐controlled and filtered to obtain clean reads, which were aligned to the mouse reference genome. Gene expression was quantified using RSEM. Differentially expressed genes were identified using |log_2_FC| ≥ 1 and Padj < 0.05. Volcano plots and heatmaps were generated using ggplot2 and pheatmap in R. GO and KEGG enrichment analyses were performed using clusterProfiler, with Padj ≤ 0.05 considered significant. GSEA was performed based on MSigDB gene sets, and ssGSEA was used to calculate immune cell infiltration scores, which were visualized as heatmaps.

### 4.34 In Vivo Targeting Analysis of FBOA‐tFNA

Female C57BL/6 mice aged 6–8 weeks and female BALB/c nude mice aged 6–8 weeks were purchased from Hangzhou Ziyuan Laboratory Animal Technology Co., Ltd. (animal production license No. SCXK [Zhe] 2024‐0004). Animal experiments were approved by the Experimental Animal Ethics Committee of Fujian Maternity and Child Health Hospital, AEC‐SFY‐XM2026‐166, and were conducted in strict accordance with institutional animal care.

To evaluate tumor‐targeted accumulation and mutation‐responsive activation of FBOA‐tFNA in vivo, UWB1.289 subcutaneous xenografts in nude mice and ID8 and ID8‐BRCA1‐mut subcutaneous tumor models in C57BL/6 mice were established. When tumors reached approximately 100 mm^3^, mice were randomly assigned to four groups: PBS, FBO‐tFNA, FBA‐tFNA, and FBOA‐tFNA. The corresponding formulations were administered by tail vein injection at 5 mg kg^−^^1^ olaparib or adavosertib equivalent. Cy5 and FAM fluorescence signals were acquired at 0, 1, 2, 4, 8, and 12 h after injection using an IVIS Spectrum small‐animal imaging system (PerkinElmer). At 12 h, mice were sacrificed, and tumors, hearts, livers, spleens, lungs, kidneys, and draining lymph nodes were collected for ex vivo imaging. A portion of each tumor was digested into single‐cell suspensions, and Cy5^+^FAM^+^ double‐positive tumor cells were analyzed by flow cytometry to validate intratumoral uptake and mutation‐responsive activation.

### 4.35 Establishment of Mouse Ovarian Cancer Models

For the syngeneic subcutaneous ovarian tumor model, logarithmic‐phase ID8‐BRCA1‐mut cells were collected, washed with PBS, and resuspended in serum‐free DMEM at 1 × 10^7^ cells mL^−^^1^. Female C57BL/6 mice aged 6–8 weeks were subcutaneously inoculated in the right dorsal flank with 100 µL cell suspension, corresponding to 1 × 10^6^ cells per mouse. When tumor volumes reached approximately 100 mm^3^, mice were randomly assigned to PBS, FBO‐tFNA, FBA‐tFNA, and FBOA‐tFNA groups, with five mice per group. Treatments were administered by tail vein injection once every 3 days for five doses. All tFNA formulations were dosed at 5 mg kg^−^^1^ for the corresponding drug equivalent: FBO‐tFNA, olaparib equivalent; FBA‐tFNA, adavosertib equivalent; and FBOA‐tFNA, olaparib and adavosertib equivalents. The PBS group received an equivalent volume of PBS. Tumor length, width, and body weight were measured every 3 days. Body weight was monitored throughout the study and expressed as the percentage change from baseline. No animal experienced a body‐weight loss exceeding 15% of its initial body weight during the study. Tumor volume was calculated as length × width^2^/2.

For the peritoneal dissemination model, ID8‐BRCA1‐mut‐Luc cells were collected and adjusted to 5 × 10^6^ cells/mL. A total of 200 µL of cell suspension, corresponding to 1 × 10^6^ cells, was intraperitoneally injected into 6‐8‐week‐old female C57BL/6 mice. Four weeks after intraperitoneal inoculation, intraperitoneal tumor formation was confirmed using IVIS imaging. Mice were then randomly assigned to PBS, free olaparib plus adavosertib, and FBOA‐tFNA groups, with five mice per group. Treatments were administered by tail vein injection once every 3 days for five doses. Olaparib and adavosertib were administered at 5 mg kg^−1^ each, either as a free‐drug combination or as FBOA‐tFNA drug equivalents. The PBS group received an equivalent volume of PBS. During treatment, in vivo imaging was performed weekly to monitor intraperitoneal tumor bioluminescence signals, ascites formation, and survival.

For the anti‐PD‐1 combination model, an ID8‐BRCA1‐mut‐Luc subcutaneous tumor model was established. When tumors reached approximately 100 mm^3^, mice were randomly assigned to PBS, FBOA‐tFNA, anti‐PD‐1 antibody, and combination therapy groups, with five mice per group. FBOA‐tFNA was administered by tail vein injection once every 3 days for five doses at 5 mg kg^−1^ olaparib and adavosertib equivalents. Anti‐PD‐1 antibody (BioXCell, BE0146, RRID: AB_10949053) was administered intraperitoneally at 10 mg kg^−^^1^ once every 3 days for five doses. In the combination group, anti‐PD‐1 was injected 24 h after FBOA‐tFNA administration. The PBS group received an equivalent volume of PBS. Tumor volume and body weight were monitored regularly. On day 28, mice were sacrificed, tumors were excised and weighed, and spleens, tumor‐draining lymph nodes, and tumors were collected for immunological and histological analyses.

Throughout the study, tumor burden did not exceed 10% of the animal's body weight, tumor volume did not exceed 2,000 mm^3^, and the maximum tumor diameter did not exceed 20 mm. Animals were monitored regularly according to the approved institutional humane‐endpoint criteria.

### 4.36 In Vivo Repeated‐Dose Tolerability Evaluation

For repeated‐dose tolerability, FBOA‐tFNA was administered intravenously every 3 days for five doses; mice were sacrificed on day 3 after the final administration. Orbital venous blood and major organs, including heart, liver, spleen, lung, and kidney, were collected. Whole blood was kept at room temperature for 30 min and centrifuged at 3000 × g for 15 min at 4°C to obtain serum. Serum aspartate aminotransferase (AST), alanine aminotransferase (ALT), UREA, creatinine (CREA), creatine kinase (CK), and lactate dehydrogenase (LDH) were measured using an automated biochemical analyzer to evaluate liver and kidney function and tissue injury. Major organs were fixed in 4% paraformaldehyde, paraffin‐embedded, sectioned, and stained with hematoxylin and eosin (H&E). Sections were examined under an optical microscope for structural damage and inflammatory changes. Serum IL‐6 and TNF‐α levels were also measured by ELISA to assess systemic inflammation or immune activation induced by FBOA‐tFNA.

### 4.37 Histopathological, Immunohistochemical, and Immunofluorescence Analyses

At the end of tumor monitoring, mice were sacrificed, and tumors and major organs were collected and fixed in 4% paraformaldehyde, paraffin‐embedded, and sectioned at 4 µm. Major organs were subjected to H&E staining. For γ‐H2AX immunohistochemistry, tumor sections were subjected to antigen retrieval, blocked, incubated with anti‐γ‐H2AX antibody (HUABIO, ET1602‐2, 1:200), followed by an HRP‐conjugated secondary antibody, and developed with DAB. For p‐STING and PD‐L1 immunofluorescence, sections were incubated with anti‐p‐STING (CST, #62912, 1:100) or anti‐PD‐L1 (Nature Biosciences, A78758, 1:200), followed by appropriate fluorophore‐conjugated secondary antibodies and DAPI counterstaining. Tumor apoptosis was detected using a TUNEL assay kit (Roche, 11684817910) according to the manufacturer's instructions, with DAPI used for nuclear counterstaining. Images were acquired using a digital slide scanning system (KF‐FL‐005, Jiangfeng). p‐STING and PD‐L1 were quantified by mean fluorescence intensity, whereas γ‐H2AX and TUNEL were quantified by positive staining area. Multiple non‐overlapping fields from five independent tumors per group were analyzed, and each tumor was treated as one biological replicate.

### 4.38 Flow Cytometric Analysis of Tumor‐Infiltrating and Peripheral Immune Cells

Flow cytometry was used to analyze immune cell composition and activation in tumor tissues, spleens, and tumor‐draining lymph nodes from ID8‐BRCA1‐mut tumor‐bearing mice. At the end of tumor monitoring, tumors, spleens, and inguinal draining lymph nodes were excised.

Tumors were minced and digested in RPMI 1640 medium (Shanghai Life‐iLab Biotech Co., Ltd, AC01L064) containing 1 mg mL^−1^ collagenase IV and 0.1 mg mL^−1^ DNase I at 37°C for 30 min. Digested suspensions were filtered through 40 µm cell strainers to obtain single‐cell suspensions. Spleens were mechanically dissociated, filtered through 40 µm strainers, and treated with ACK red blood cell lysis buffer (NCM Biotech, C100C3). Draining lymph nodes were mechanically dissociated and filtered directly. Cells were washed with PBS, resuspended, and stained with fluorochrome‐conjugated antibodies according to the experimental design.

For intratumoral T‐cell infiltration, cells were stained with anti‐CD3‐FITC, anti‐CD4‐PE, and anti‐CD8‐APC to quantify CD3^+^CD4^+^ and CD3^+^CD8^+^ T cells and calculate the CD4^+^/CD8^+^ ratio. Splenic activated CD8^+^ T cells (CD3^+^CD8^+^CD69^+^) were detected using anti‐CD3‐FITC, anti‐CD8‐APC, and anti‐CD69‐PE. Mature DCs (CD11c^+^CD80^+^CD86^+^) were detected using anti‐CD11c‐FITC, anti‐CD80‐PE, and anti‐CD86‐APC. NK cells (CD45^+^CD3^−^NK1.1^+^) were detected using anti‐CD45‐APC‐A750, anti‐CD3‐FITC, and anti‐NK1.1‐APC. CD8^+^IFN‐γ^+^ effector T cells in tumor‐draining lymph nodes were detected using anti‐CD3‐FITC, anti‐CD8‐APC, and anti‐IFN‐γ‐PE. PD‐1 expression on CD8^+^ T cells in tumor tissues or ascites was detected using anti‐CD3‐FITC, anti‐CD8‐APC, and anti‐PD‐1‐PE. In the anti‐PD‐1 combination model, exhausted‐like and effector memory precursor CD8^+^ T‐cell subsets were analyzed using anti‐CD3‐FITC, anti‐CD8‐APC, anti‐PD‐1‐PE, and anti‐CD44‐PC5.5 to evaluate CD3^+^CD8^+^PD‐1^+^CD44^+^ and CD3^+^CD8^+^PD‐1^−^CD44^+^ populations. Splenic memory T‐cell subsets were analyzed using anti‐CD3‐FITC, anti‐CD8‐APC, anti‐CD44‐PC5.5, and anti‐CD62L‐PE to evaluate central memory T cells (CD3^+^CD8^+^CD44^+^CD62L^+^) and effector memory T cells (CD3^+^CD8^+^CD44^+^CD62L^−^). CD8^+^IFN‐γ^+^ effector T cells in tumor‐draining lymph nodes were analyzed using anti‐CD3‐FITC, anti‐CD8‐APC, and anti‐IFN‐γ‐PE. For ex vivo intracellular IFN‐γ staining, cells were incubated with a protein transport inhibitor to block cytokine secretion, without additional PMA/ionomycin stimulation, followed by fixation/permeabilization and staining with anti‐IFN‐γ‐PE.

Flow cytometry antibodies were purchased from the indicated commercial suppliers. When ex vivo maintenance of NK cells was required, cells were cultured in NK Cell Basal Medium Kit (XR Bio, Hangzhou, China, XRNKSFM‐1‐1000) according to the manufacturer's instructions. Cells were incubated with antibodies at 4°C in the dark for 30 min, washed with PBS, and resuspended. Data were acquired using a Beckman CytoFLEX Flow Cytometer and analyzed with FlowJo. Debris was excluded based on FSC‐A/SSC‐A, and doublets were excluded using FSC‐H versus FSC‐W plots. Immune cell subsets and activation status were analyzed within the singlet population.

### 4.39 Statistical Analysis

All quantitative data are presented as mean ± standard deviation (SD) unless otherwise indicated. Statistical analyses were performed using GraphPad Prism software. For comparisons between two groups, t‐test analysis was performed as appropriate. For comparisons among multiple groups, one‐way or two‐way analysis of variance (ANOVA) followed by appropriate post hoc tests was performed, as indicated by the experimental design. Sample sizes and biological replicates were determined according to the requirements of each experiment and are indicated in the corresponding figures, figure legends, or Methods descriptions where applicable. A value of p < 0.05 was considered statistically significant.

## Author Contributions

Conception and design: C.W., M.Y., C.L., and P.S.; Chemical, physical, and biological experiments were carried out by C.W., M.Y., J.X., D.W., and Y.S.; Collection of patient‐derived ovarian tumor specimens: X.M.; Analysis and interpretation of data: C.W. and M.Y.; Writing, review, and editing of the manuscript: C.W. and P.S.; The work was supervised by C.L. and P.S.; All authors have given approval to the final version of the manuscript.

## Funding

This work was supported by grants from the Fujian Provincial Traditional Chinese Medicine Science and Technology Project Funding Program (No. 2026QNGGB00 9); the National Natural Science Foundation of China (No. 82674160); the Fujian Provincial Natural Science Foundation (Nos. 2026J0011280 and 2026J002019); the Fujian Provincial Medical Peak Excellence Initiative; and the 2025 Science and Technology Innovation Start‐up Fund of Fujian Maternity and Child Health Hospital (Research Fund for Introduced Talents, Fuyou YCXY25‐04).

## Ethics Statement

The use of patient‐derived ovarian cancer organoids in this study was approved by the Ethics Committee of Fujian Maternity and Child Health Hospital (Approval No. 2026KY166‐01). Written informed consent was obtained from the patient before sample collection, and all samples were de‐identified before use. This study was carried out in accordance with the relevant guidelines and regulations for the care and use of laboratory animals. All the animal procedures were approved by the Experimental Animal Ethics Committee of Fujian Maternity and Child Health Hospital (Approval No. AEC‐SFY‐XM2026‐166).

## Conflicts of Interest

The authors declare no conflicts of interest.

## Supporting information




**Supporting File**: advs78457‐sup‐0001‐SuppMat1.docx.

## Data Availability

The data that support the findings of this study are available from the corresponding author upon reasonable request.
