# Supplementary material for: BRCA1 Mutation‐Recognizing Tetrahedral Framework Nucleic Acid Nanoplatform Enables Coordinated PARP/WEE1 Inhibition and STING‐Associated Antitumor Immunity in Ovarian Cancer
Source: Adv Sci (Weinh). 2026 Oct 28:e78457. Online ahead of print. doi: 10.1002/advs.78457 (PMC13717119; doi:10.1002/advs.78457)
Supplement: Supplementary file 1 [file ADVS-9999-e78457-s001.docx]

Supporting Information

**BRCA1 Mutation-Recognizing Tetrahedral Framework Nucleic Acid**  **Nanoplatform Enables Coordinated PARP/WEE1 Inhibition and STING-Associated Antitumor Immunity in Ovarian Cancer**

*Chenyi Wang^1,2,3,4,5,^**^‡^,* *Mengying Yao^5,‡^, Jinhui Xie**^1,2,3,4^, Dingjie Wang^1,2,3^,* *Yashi Shi^1,2,3^, Xiaodan Mao^1,2,3^, Chunhua Lu**^4,5,^*, and* *Pengming Sun^1,2,3,4,^**

^1^Laboratory of Gynecologic Oncology, Fujian Maternity and Child Health Hospital, College of Clinical Medicine for Obstetrics & Gynecology and Pediatrics, Fujian Medical University, Fuzhou 350001, China;

^2^Fujian Clinical Research Center for Gynecological Oncology, Fujian Maternity and Child Health Hospital (Fujian Obstetrics and Gynecology Hospital), Fuzhou 350001, China;

^3^Fujian Key Laboratory of Women and Children’s Critical Diseases Research, Fuzhou 350001, China;

^4^School of Medicine, Fuzhou University, Fuzhou 350108, China;

^5^MOE Key Laboratory for Analytical Science of Food Safety and Biology, Fujian Key Laboratory of Analysis and Testing Technology for Food Safety and Health, College of Chemistry, Fuzhou University, Fuzhou 350108, China

^‡^These authors contributed equally: Chenyi Wang and Mengying Yao.

**Corresponding Authors**

*Chunhua Lu, Ph.D., Prof., E-mail: chunhualu@fzu.edu.cn.

*Pengming Sun, Ph.D., Prof., E-mail: fmsun1975@fjmu.edu.cn.

**Figure S1. Oligonucleotide backbone sequences and topological designs of the functionalized modification modules implemented in this study. (A)** Core single-stranded sequences (S1-S4) constituting the tetrahedral framework nucleic acid (tFNA) scaffold. The regions highlighted in identical colors represent local complementary base-pairing domains engineered to drive the topological self-assembly of the framework. **(B)** Folate-targeted single-stranded sequence integrated at vertex 1 (V1), engineered to endow the nanoplatform with active recognition and specific endocytic internalization capabilities toward folate receptor-α (FR-α) overexpressing tumor cells. **(C)** BRCA1 mutation-specific molecular beacon sequences integrated at vertex 2 (V2). Illustrated are the molecular beacon designed to recognize the BRCA1 2594delC-associated mutant transcript in human UWB1.289 cells, the corresponding scrambled molecular beacon (scrMB) used as a nonresponsive sequence control, and the molecular beacon designed to recognize the BRCA1 972delG-associated mutant transcript in murine ID8-BRCA1-mut cells. **(D)** Reduction-responsive olaparib prodrug module immobilized at vertex 3 (V3), comprising the olaparib-specific release strand, protective strand, and olaparib drug-linker arm containing a redox-responsive disulfide bond. **(E)** Reduction-responsive adavosertib prodrug module immobilized at vertex 4 (V4), comprising the adavosertib-specific release strand, protective strand, and adavosertib drug-linker arm containing a redox-responsive disulfide bond.

**Figure S2.** **(A)** Synthesis of the self-assembling anchor unit for the folate-mediated active targeting module (Folate-PEG₂K-ssDNA). **(B)** Stepwise chemical synthesis route of the reduction-responsive olaparib nucleic acid prodrug (Olaparib-SS-ssDNA).

**Figure S3.** Stepwise chemical synthesis route of the reduction-responsive adavosertib nucleic acid prodrug (Adavosertib-SS-ssDNA).

**Figure S4. Characterization profiles of various functionalized modification modules and key synthetic intermediates. (A)** NMR spectrum of the folate-polyethylene glycol active ester intermediate (FA-PEG_2_K-NHS). **(B)** Mass spectrum (MS) of the olaparib propylamine intermediate (Olaparib-C_3_-NH_2_). **(C)** Mass spectrum of the redox-responsive disulfide-linked olaparib derivative (Olaparib-SS-COOH). **(D)** Mass spectrum of the terminally activated maleimide-modified olaparib prodrug (Olaparib-SS-MAL). **(E)** Mass spectrum of the Boc-protected alkylated adavosertib intermediate (Adavosertib-NHBoc). **(F)** Mass spectrum of the terminal maleimide-modified adavosertib prodrug (Adavosertib-SS-MAL).

**Figure S5.** **HPLC and LC–MS/MS characterization of drug loading and drug-species distribution, together with quantitative analysis of BRCA1 and FOLR1 expression. (A)** HPLC chromatograms of olaparib standards at different concentrations. **(B)** Standard calibration curve of olaparib with the linear regression equation Y = 68517X + 71607 and R² = 0.9998. **(C)** Representative HPLC chromatogram of free olaparib in the ultrafiltration filtrate, with a retention time of approximately 6.319 min. **(D)** HPLC chromatograms of adavosertib standards at different concentrations. **(E)** Standard calibration curve of adavosertib with the linear regression equation Y = 60385X + 31830 and R² = 0.9999. **(F)** Representative HPLC chromatogram of free adavosertib in the ultrafiltration filtrate, with a retention time of approximately 6.768 min. **(G)** Loading efficiencies of olaparib and adavosertib from three independently prepared nanoplatform batches; each point represents one batch. **(H)** Drug molecules incorporated per tFNA as a function of drug input, showing near-stoichiometric incorporation of both olaparib and adavosertib at higher input equivalents. **(I)** Densitometric quantification of BRCA1 and FOLR1 expression corresponding to Figure 1Q in HeLa, SK-OV-3, UWB1.289, and UWB1.289-BRCA1-restored cells. Protein levels were normalized to β-actin and expressed relative to HeLa cells. **(J)** Representative LC–MS/MS chromatograms distinguishing free and linker-bound olaparib and adavosertib under PBS or GSH conditions.

**(K)** Relative distribution of free and linker-bound drug species calculated from LC–MS/MS peak-area integration. Statistical significance was evaluated using one-way ANOVA (I).

**Figure S6. Flow cytometric validation of FOLR1-associated cellular uptake and scrambled-beacon control of BRCA1 mutant-transcript-specific recognition.** **(A)** Flow cytometric analysis of surface FOLR1 expression in HeLa, SK-OV-3, UWB1.289, and UWB1.289-BRCA1-restored cells. **(B)** Flow cytometric analysis of Cy5-labeled FBOA-tFNA uptake in the indicated cell models. **(C)** Quantification of Cy5 fluorescence intensity (MFI) corresponding to the cellular uptake analysis in (B). **(D)** Folate competition assay in UWB1.289 cells showing Cy5 fluorescence following treatment with PBS, FBOA-tFNA, or FBOA-tFNA in the presence of excess free folic acid.  **(E)** Representative confocal images of UWB1.289 and UWB1.289-BRCA1-restored cells treated with F-scrMB-O-tFNA or FBO-tFNA. Cy5 indicates nanoparticle-associated cellular uptake, FAM indicates molecular-beacon activation, and Hoechst labels nuclei. **(F–G)** Quantification of Cy5 and FAM fluorescence intensities, respectively. **(H)** Cell viability following the indicated treatments. Statistical significance was evaluated using one-way ANOVA (F-H).

**Figure S7.** Densitometric quantification of WEE1, p-Chk1(Ser345), p-CDK1(Tyr15), and γ-H2AX protein expression corresponding to Figure 2R. Protein levels were normalized to β-actin and expressed relative to the control group. Statistical significance was evaluated using t-test analysis.

**Figure S8.** **(A-B)** Schematic illustration of the modular assembly of FBOA-tFNA (A) and its proposed mechanism involving coordinated PARP/WEE1 inhibition, DNA damage accumulation, checkpoint abrogation, mitotic catastrophe, and synthetic lethality (B). **(C)** Native PAGE analysis of the stepwise, hierarchical self-assembly of FBOA-tFNA. Lane samples: partial hybridization intermediates and the intact FBOA-tFNA nanoparticle. **(D-F)** Physicochemical characterization of FBOA-tFNA. (D) Dynamic light scattering analysis of the hydrodynamic diameter distribution of tFNA and FBOA-tFNA. (E) Polydispersity index (PDI) of tFNA and FBOA-tFNA. (F) Zeta potential of tFNA and FBOA-tFNA. **(G-J)** Structural stability and robustness of FBOA-tFNA under physiological conditions. (G) Native PAGE analysis of FBOA-tFNA co-incubated with DNase I (10 U/mL) at 37 °C for 0-12 h. (H) Statistical quantification of the relative band grayscale intensity from native PAGE analysis following DNase I challenge. (I) Native PAGE analysis of FBOA-tFNA incubated in 10% fetal bovine serum (FBS) at 37 °C for 0-12 h. (J) Statistical quantification of the structural integrity based on band grayscale intensity following serum incubation. **(K)** Schematic illustration of the recombinant lentiviral expression vector constructed to introduce the signature truncated BRCA1 mutation (972delG) for the establishment of the stable ID8-BRCA1-mut cell model. Statistical significance was evaluated using one-way ANOVA (H, J).

**Figure S9. Quantitative evaluation of PARP/WEE1 synergy, intracellular dual-drug coordination, and reduction-responsive drug release.** **(A)** Bliss synergy heatmap for free olaparib and adavosertib at indicated IC₅₀-based combinations. **(B)** Bliss synergy heatmap for FBO-tFNA and adavosertib at indicated IC₅₀-based combinations. **(C-D)** Intracellular olaparib- and adavosertib-associated levels after treatment with free drugs, separate carriers, or FBOA-tFNA. **(E)** Intracellular olaparib/adavosertib molar ratio over time. **(F)** Intracellular olaparib-associated levels following free olaparib or FBO-tFNA treatment. **(G–H)** Cumulative release of olaparib and adavosertib from cleavable and non-cleavable constructs in PBS or 10 mM GSH.

**Figure S10. Schematic design of the engineered partial BRCA1 construct containing the 2594delC-equivalent mutant sequence.** The construct includes an N-terminal 4×FLAG tag, GS linker, and partial BRCA1-derived coding sequence under the CMV promoter. The corresponding wild-type and mutant sequences within the molecular-beacon recognition region are shown, together with the transcript-specific annotation NM_001407571.1:c.2262del. The construct encodes a 1,047-bp coding sequence with a predicted 348-aa product of approximately 39.1 kDa.

**Figure S11. (A)** Serum biochemical analyses of nude mice after different treatments. Levels of creatine kinase (CK) and lactate dehydrogenase (LDH) were measured to assess systemic toxicity. **(B)**  Representative hematoxylin and eosin (H&E) staining images of heart, liver, spleen, lung, and kidney tissues collected from UWB1.289 tumor-bearing nude mice treated with PBS (G1), FBO-tFNA (G2), FBA-tFNA (G3), and FBOA-tFNA (G4). Scale bar: 100 μm. Statistical significance was evaluated using one-way ANOVA (A).

**Figure S1**2**. (A)** Serum biochemical analyses of C57BL/6 mice after different treatments. Levels of aspartate aminotransferase (AST), alanine aminotransferase (ALT), urea (UREA), creatinine (CREA), creatine kinase (CK), and lactate dehydrogenase (LDH) were measured to assess systemic toxicity. **(B)**  Representative hematoxylin and eosin (H&E) staining images of heart, liver, spleen, lung, and kidney tissues collected from ID8-BRCA1-mut tumor-bearing C57BL/6 mice treated with PBS (G1), FBO-tFNA (G2), FBA-tFNA (G3), and FBOA-tFNA (G4). Scale bar: 100 μm. Statistical significance was evaluated using one-way ANOVA (A).

**Figure S13. GO and KEGG enrichment analyses of differentially expressed genes after FBOA-tFNA treatment. (A)** KEGG pathway enrichment bar plot of upregulated differentially expressed genes after FBOA-tFNA treatment. **(B)** KEGG pathway enrichment bar plot of downregulated differentially expressed genes after FBOA-tFNA treatment. **(C)** GO functional classification analysis of downregulated differentially expressed genes after FBOA-tFNA treatment. **(D)** GO enrichment bubble plot of downregulated differentially expressed genes after FBOA-tFNA treatment.

**Figure S14. GSEA of representative gene sets after FBOA-tFNA treatment. (A-D)** GSEA plots showing enrichment of gene sets associated with lysosome organization (A), autophagy (B), DNA replication checkpoint (C), and Toll-like receptor signaling pathway (D) after FBOA-tFNA treatment.

**Figure S15. Pharmacological STING inhibition attenuates FBOA-tFNA-induced STING signaling and dendritic-cell maturation.** **(A)** Western blot analysis of STING, TBK1, and IRF3 phosphorylation following FBOA-tFNA treatment with or without STING inhibitor (STINGi), with the corresponding p/total protein ratios shown. **(B)** Representative flow-cytometry plots of CD80⁺CD86⁺ mature BMDCs following the indicated treatments. **(C)** Quantification of CD80⁺CD86⁺ BMDCs. Statistical significance was evaluated using one-way ANOVA (C).

**Figure S16. Flow cytometric gating strategies for BMDC maturation and T-cell activation assays. (A)** Gating strategy for CD11c⁺CD80⁺CD86⁺ mature BMDCs. **(B)** Gating strategy for CD3⁺ T cells expressing CD25⁺ and CD69⁺ in an independent T-cell Transwell coculture assay. **(C)** Representative flow cytometry plots of CD25⁺ T cell proportions within CD3⁺ cells across different treatment groups.

**Figure S17. (A-F)** The flow cytometric gating strategies for characterizing tumor-infiltrating helper T cells (CD3^+^CD4^+^) and cytotoxic T lymphocytes (CD3^+^CD8^+^) (A), early-activated cytotoxic T cells expressing CD69^+^ (B), mature intratumoral dendritic cells (CD11c⁺CD80⁺CD86⁺) (C), infiltrating natural killer cells (CD45⁺CD3⁻NK1.1⁺) (D), IFN-γ⁺-producing effector cytotoxic T lymphocytes (CD8^+^IFN-γ⁺) (E), and PD-1 expression on tumor-infiltrating cytotoxic T cells (F) across different treatment groups.

**Figure S18. Quantitative analysis of tumor histological and immunofluorescence markers following the indicated treatments. (A)** Quantification of p-STING mean fluorescence intensity. (B) Quantification of TUNEL-positive area. **(C)** Quantification of γ-H2AX-positive area. **(D)** Quantification of PD-L1 mean fluorescence intensity. Each point represents one independent tumor specimen. Statistical significance was evaluated using one-way ANOVA (A-D).

**Figure S19. (A-B)** The flow cytometric gating strategies for characterizing IFN-γ⁺-producing effector cytotoxic T lymphocytes (CD8^+^IFN-γ⁺) in tumor-draining lymph nodes (A) and PD-1 expression on ascites-infiltrating cytotoxic T cells (B) from divergent treatment cohorts.

**Figure S20.** **(A-C)** The flow cytometric gating strategies for characterizing PD-1^+^ and CD44^+^ expression profiles on tumor-infiltrating CD3^+^CD8^+^ T cells (A), CD62L^+^ and CD44^+^ memory differentiation lineages on splenic T cells (B), and IFN-γ⁺-producing effector cytotoxic T lymphocytes (CD8^+^IFN-γ⁺) in tumor-draining lymph nodes (C) from divergent treatment cohorts.
